# *Methanobrevibacter* attenuation via probiotic intervention reduces flatulence in adult human: A non-randomised paired-design clinical trial of efficacy

**DOI:** 10.1371/journal.pone.0184547

**Published:** 2017-09-22

**Authors:** Minseok Seo, Jaeyoung Heo, Joon Yoon, Se-Young Kim, Yoon-Mo Kang, Jihyun Yu, Seoae Cho, Heebal Kim

**Affiliations:** 1 C&K Genomics, Seoul National University Research Park, Seoul, Republic of Korea; 2 Department of Animal Biotechnology, Chonbuk National University, Jeonju, Republic of Korea; 3 Interdisciplinary Program in Bioinformatics, Seoul National University, Seoul, Republic of Korea; 4 R&D Center, CTCBIO, Inc., Hwaseong-si, Gyeonggi-do, Republic of Korea; 5 Department of Agricultural Biotechnology, Seoul National University, Seoul, Republic of Korea; 6 Research Institute of Agriculture and Life Sciences, Seoul National University, Seoul, Republic of Korea; UCLA, UNITED STATES

## Abstract

**Trial design:**

The aim of this study was to investigate which of the gut microbes respond to probiotic intervention, as well as study whether they are associated with gastrointestinal symptoms in a healthy adult human. For the experimental purpose, twenty-one healthy adults were recruited and received probiotic mixture, which is composed of five *Lactobacilli* strains and two *Bifidobacteria* strains, once a day for 60 days. Defecation survey and Bioelectrical Impedance Analysis were conducted pre- and post-administration to measure phenotypic differences. Stool samples of the subjects were collected twice.

**Methods:**

The statistical analysis was performed for pair designed metagenome data with 11 phenotypic records of the bioelectrical impedance body composition analyzer and 6 responses of the questionnaires about gastrointestinal symptom. Furthemore, correlation-based network analysis was conducted for exploring complex relationships among microbiome communities.

**Results:**

The abundances of *Citrobacter*, *Klebsiella*, and *Methanobrevibacter* were significantly reduced, which are strong candidates to be highly affected by the probiotic administration. In addition, interaction effects were observed between flatulence symptom attenuation and decreasing patterns of the *Methanobrevibacter* abundance.

**Conclusions:**

These results reveal that probiotic intervention modulated the composition of gut microbiota and reduced the abundance of potential pathogens (i.e. *Citrobacter* and *Klebsiella)*. In addition, methanogens (i.e. *Methanobrevibacter*) associated with the gastrointestinal symptom in an adult human.

## Introduction

Probiotics are widely consumed dietary supplements composed of single or combination of viable microbial strains providing benefits to human health. The genera such as *Lactobacilli* and *Bifidobacteria* have been mostly circulated in the market based on their health-promoting mechanisms of physical, biochemical, and immunomodulatory interactions with gut microbiota and host physiology in the gastrointestinal tract in animal models [1, 2]. Several clinical studies suggest the efficacy of probiotics on gastrointestinal symptoms such as constipation, diarrhea, irregular bowel movement, incomplete bowel movement, flatulence and abdominal pain in normal or disease-state subjects [3, 4]. Further studies have reported the effectiveness in reducing the risks of irritable bowel syndrome (IBS), inflammatory bowel disease (IBD), autoimmune disease, pathogen infections and colon cancer in animals and humans [5, 6]. Of note, health-promoting effects of probiotics are strain-specific or combination-specific; their effects on other strains or combinations are not yet known [7]. These claims related to gastrointestinal symptoms and diseases lack reproducibility and mechanistic evidence in humans.

The gut microbiota plays a crucial role in host physiology, metabolism, immune system, and gastrointestinal infectious diseases [8, 9]; influenced by many environmental factors such as host, dietary habits, aging, and antimicrobial drugs, leading to considerable differences in its composition among subjects [10–14]. Further evidence supports that changes in gut microbiota modifies immune responses, inflammation, and insulin resistance, including their related metabolic syndromes, in animals [15]. The healthy gut microbiota is known to provide defense mechanisms to gut barrier against invading pathogens and indigenous pathobionts, preventing the disruption of homeostasis of the normal microbial ecosystem in an individual [16]. Although the studies with culture-independent methods (i.e. qPCR, DGGE) suggest the use of some probiotic products changed the abundance of indigenous gut microbes in healthy subjects [7], the beneficial roles of particular probiotics for the general public are not entirely understood.

The advance in sequencing technique makes it possible to overcome the limitation that microbiome can be identified solely depending on the cultivation. Due to this reason, a simultaneous identification of the diverse microbiomes using next generation sequencing (NGS), the so-called ‘metagenome analysis’, was widely used to detect the unculturable microbiomes and to quantify their relative abundances. Since diverse species, of microbiomes, are sequenced simultaneously, one of the important issues in the metagenome approach is distinguishing the microbial taxa. To accurately separate diverse taxa, well-known phylogenetic markers, which are highly conserved within species but distinguishable between species, should be employed [17–19]. From a large number of studies, ribosomal RNAs (rRNAs) have been discovered as a viable marker, especially the 16s rRNA [20, 21]. Furthermore, the recently developed Illumina’s Miseq can identify up to 500 bp fragment, which provides practically useful resolution in genus level since 500 bp is long enough to cover V1-V2 or V3-V4 variable regions [22]. For these reasons, microbial community change can be practically measured in diverse experimental conditions.

In this study, we aimed to investigate gut microbial changes in response to probiotic intervention consisted of *Lactobacilli* and *Bifidobacteria*, as well as whether they are associated with gastrointestinal symptoms and subject’s phenotypes in an adult human. Our study illustrates that probiotic intervention modulated the composition of gut microbiota and reduced the abundance of potential pathogens, such as *Citrobacter* and *Klebsiella*, and methanogens like *Methanobrevibacter* associated with the gastrointestinal symptom in an adult human.

## Materials and methods

### Ethics approval statement

This study was performed in accordance with the Institutional Review Board of Seoul National University (Korea; SNU IRB No.1507/002-012). The study was approved by the Ethical Committee of the SNU IRB (Seoul, Korea), and informed consent were obtained from all 21 volunteers before enrollment in the study. Sampling and all subsequent steps described in the Materials and Methods have been conducted in accordance with the approved guidelines (S1 File). In addition, we misapprehended the international protocol on human probiotics trial, and proceeded the study with an IRB approval. After reviewing the guidelines, the clinical trial was retrospectively registered (2016-08-12) and approved in Clinical Research Information Service (CRIS) (KCT0002008) (S2 File).

### Experimental design for metagenome study

This study’s sample size was determined based on the published work that uses 20 subjects to measure the effects of probiotic mixtures in the gut microbiome [23]. This study investigated the effect of probiotic mixture at three time points; before intervention, 2-month intervention, and a year of intervention. We also recruited our participants in accordance with Venturi’s protocol, and collected samples from 20~25 individuals, in case of failure to collect stool samples from any of the individuals. Twenty-one healthy adult volunteers were randomly recruited through bulletin announcements and had given their informed consent in Seoul, South Korea. Of the volunteers, 13 individuals belong to six families (2~3 per family). All participants All 21 subjects received a probiotic mixture (PROBA^®^, 525 mg) once a day for 60 days, obtained in person from CTCBIO, Inc., Seoul, Korea. A PROBA® capsule contained 20 billion viable lyophilized bacteria. The probiotic mixture for PROBA® (PROBA Formula) consisted of 6 species of probiotics (*L*. *plantarum*, *L*. *salivarius*, *L*. *casei*, *L*. *acidophilus*, *B*. *animalis subsp*. *lactis* and *B*. *bifidum*). Stool samples of subjects were collected twice (before and after 60 days of probiotic administration) in sterile plastic containers and stored at -80°C. The survey and fecal microbial community data from nineteen subjects (10 males and 9 females) were carried forward for further analysis, dropping two from the original 21 participants. The stool sample of one subject did not pass the quality control test of 16s rRNA sequencing analyses; the other subject withdrew from the study for a personal non-medical reason. One subject (Sample No: 1) declared his use of antibiotics before the after-trial measurement; the before-trial measurement is not affected.

### Stool DNA preparation and microbial community analysis

In this study, there are three measured outcomes such as abundances of the gut microbiomes derived from feces, Bioelectrical Impedance Analysis, and Survey of gastrointestinal symptoms. For measuring primary outcome, abundances of the gut microbiomes, stool samples were collected as following steps. Stool DNA was isolated using Epicentre DNA isolation kits. Approximately 900ng of DNA were extracted from each sample. DNA quality was confirmed by a Bioanalyzer using an Agilent RNA 6000 Pico Kit (Agilent, Santa Clara, CA). All the samples from the reservoir were prepared using the 16S library preparation protocol and the Nextera XT DNA index kit (Illumina, San Diego, CA) to target the V3-V4 variable regions of the 16S rRNA gene. Quantification of the library was measured by real-time PCR using CFX96 real-time system (BioRad, Hercules, CA). Samples from the reservoir were loaded onto a MiSeq reagent cartridge (Illumina, San Diego, CA) and then onto the instrument. Automated cluster generation was initially performed, followed by the 2x300bp paired-end sequencing. The resulting sequence reads were equally distributed across the samples. For the setting the blind test, any information of the microbiome community did not provide to the participants.

### Metagenome analysis for quantification of operational taxonomic units

To quantify OTUs’ abundance, paired-end sequences were preprocessed following these steps: (1) Poor quality reads were filtered out and Illumina’s adapter sequences were removed by Trimmomatic v0.33 [24]; (2) Overlapped sequences were generated by performing assembly between paired-end sequence using FLASH-1.2.11 with “-m 35 -M 200 -r 300 -f 500 -s 50 -t 4” options [25]; (3) QIIME v1.9.1 was employed to detect OTUs and to quantify abundances of their [26]. First, overlapped fastq files were transformed into fasta format. Next, labeled fna files were generated using subject information with *add_qiime_labels*.*py* implemented in QIIME. From the fna files, the OUT-picking analysis was performed using *pick_open_reference_otus*.*py* in QIIME. Finally, *summarize_taxa*.*py* was used to quantify OTUs’ abundances at each taxonomic level.

### Statistical analysis to detect significantly changed OTUs between before and after trials

We statistically analyzed the samples to detect the probiotic intervention effects on microbes. Before comparing OTU’s abundances between before and after trials, trimmed mean of M values (TMM) normalization was performed in each taxonomic count data to consider different library size [27]. Using these relative abundances in each OTU, the Analysis of Deviance (ANODEV) model was employed for significance test between trials in genus, family, and phylum levels, respectively. Paired design sample was considered in this study, therefore paired test was performed using the following model:

$$\log\left(\theta_{ijk}\right)=\mu_{j}+\tau_{ij}+\beta_{jk}$$ (Eq 1)

log⁡(E(Normalizedabundance))=μ+Stage+Individual (Eq 2),

where *i* represents before and after trials, *j* is OTUs, and *k* is individual (Eq 1). To consider the paired sample design in the model, the ‘Individual’ term was included as an explanatory variable in the linear predictor as shown in (Eq 2). Finally, the negative-binomial assumption was considered as a response variable to solve the over-dispersion problem in count data. Under the null hypothesis, *H*_0_: *Stage* = 0, likelihood ratio test (LRT) was performed and probability values were adjusted by false discovery rate (FDR) multiple testing adjustment. Here, 5% significance level was considered as significant result.

### Network analysis to simultaneously investigate complex relationships between microbiome-microbiome, microbiome-trait, and trait-trait

In order to investigate complex relationships, correlation-based network analysis was employed. The employed method is well developed in transcriptome research field for detecting gene-gene interaction. In this study, this method is used to investigate not only the microbiome-microbiome interaction but also microbiome-trait and trait-trait interactions. To measure these interactions, Spearman’s correlation was used to consider many ordinal variables (i.e. six gastrointestinal symptoms). There are two categorical variables in the traits; in order to calculate their correlation coefficient, Sex (0: Female and 1: Male) and Stage (0: Before and 1: After), were coded as numerical values. A total of 10 variables were included in correlation-based network analysis: Stage, Sex, Height, Age, Constipation, Diarrhea, Aperiodicity, Incomplete bowel movement, Flatulence, and Abdominal pain. Finally, significantly detected OTUs (FDR adjusted P-value < 0.05) in the genus (*n = 16*), family (*n = 19*), and phylum (*n = 2*) taxonomic levels, were employed comparing abundances between before and after trials. A total of 47 features were used in correlation-based network analysis, and their correlation and compactness were measured in *parcor* package implemented in R [28]. Significant relationship was defined as FDR adjusted P-value < 0.05 from the Spearman’s correlation test, and the correlation matrix was visualized using *qgraph* package in R with *spring* layout in order to site node corresponding to their centrality.

### Subject information and dietary questionnaires

Subjects were asked to record all nutritional intakes for the three days before stool collection. The following six gastrointestinal symptoms were asked to the subjects before and after administration: constipation, diarrhea, irregular bowel movement, incomplete bowel movement, flatulence, and abdominal pain. The severity of symptoms are graded on a six-step scale ranging from the number (1), minimal (2), mild (3), moderate (4), severe (5), very severe, to (6) distress. To statistically test whether questionnaires’ responses differ between before and after trials, ordered logistic regression model was employed to consider ordinal response variable. Symptom relief by 60 days of probiotic intervention was statistically tested using the following model:

$${logit(Response)}_{i}=\beta_{0}+\beta_{1}{Stage}_{1i}+\beta_{2}{Sex}_{2i}+\beta_{3}{Height}_{3i}+\beta_{4}{Age}_{4i}+\beta_{5}{Family}_{5i}+\beta_{6}{ID}_{6i}$$ (Eq 3)

,where *Response* represents each questionnaire response and *Stage*_1*i*_ is before- and after- administration. In order to consider the paired sample design, ID term was included as explanatory variable along with three other covariates: Sex, height, age, and family information. The statistical test was performed on the proportional odds logistic regression model using *polr* function implemented in the *MASS* package of *R*. The square matrix was optimized via the Hessian method.

### Bioelectrical impedance analysis for investigation of probiotic effects in obesity indexes

A total of 11 indexes were measured by the bioelectrical impedance body composition analyzer (Inbody230, InBody Co. Ltd., Seoul, Korea): weight, skeletal muscle mass, body fat mass, total body water, fat-free mass, protein, mineral, body mass index (BMI), body fat percentage, waist-hip ratio, and basal metabolic rate. These indexes were measured three times in each before and after trials to consider technical errors in statistical analysis. In order to investigate the changes between before and after trials, measured indexes were statistically analyzed through the following Analysis of Covariance (ANCOVA) model (Eq 4):

$${Index}_{ijklm}=\mu +{Stage}_{i}+{Sex}_{j}+{Height}_{k}+{Age}_{l}+{Family}_{m}+{Tech}_{n}+Error(ID)+\varepsilon_{ijklmn},\varepsilon_{ijklmn}∼N(0,\sigma^{2})$$ (Eq 4),

where response variable, *Index*, represents each index, *Stage*_*i*_ is for before and after trials, and *Tech*_*n*_ represents technical replications. In addition, the three covariates: Sex, height, age, and family information were considered in the model for their potential effects on the measured indexes. In the statistical model, *Stage* term is of the main interest, therefore the statistical test was performed under the null hypothesis, *H*_0_: *Stage* = 0.

## Results

### Description of the subject information and their summary statistic in metagenome analysis

**Fig 1:**
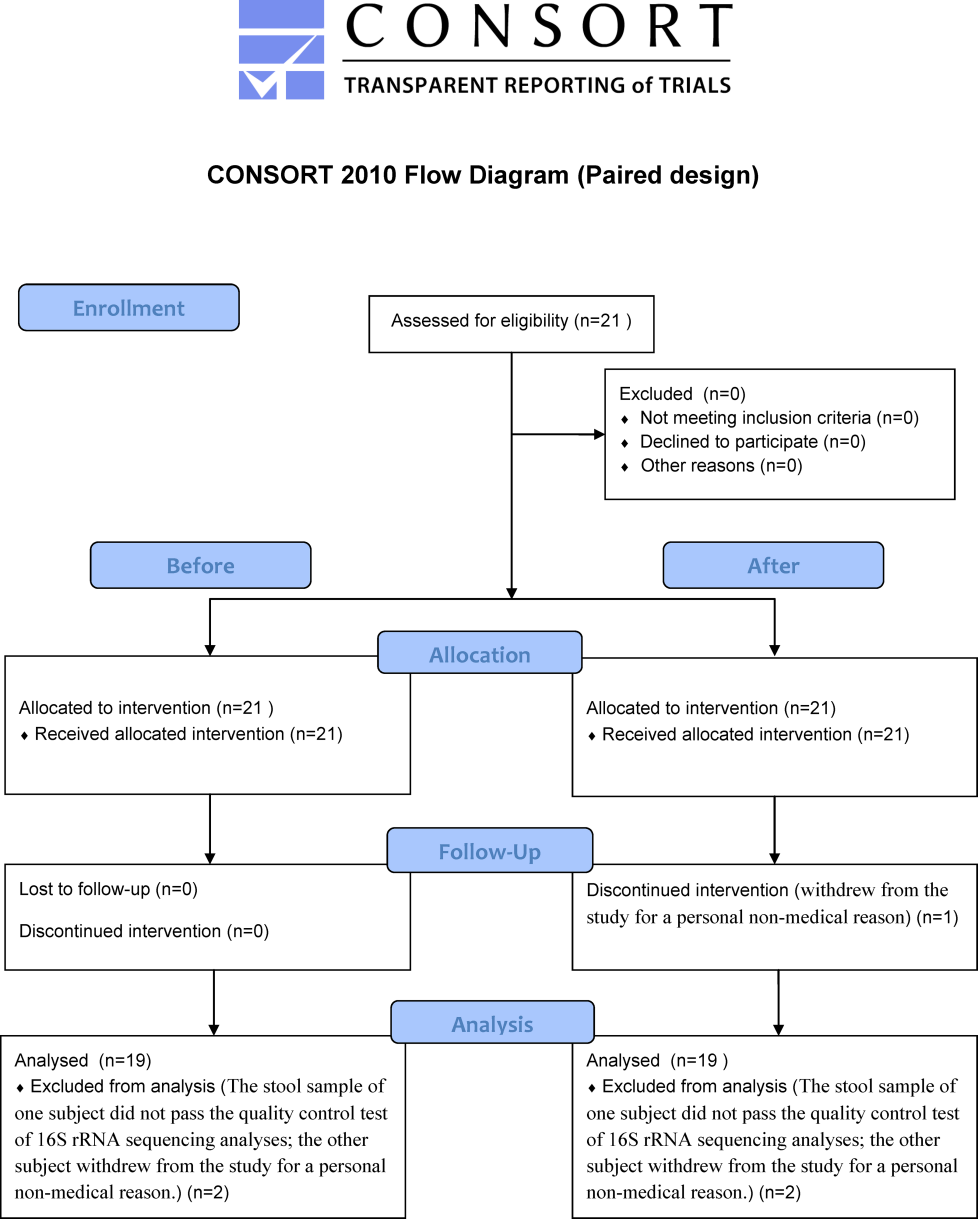
Flow diagram for paired design experiment.

From the recruited (2015-12-20 to 2016-06-30) twenty-one healthy adult volunteers (11 males and 10 females, mean age 42 years; range 20–59) (S3 File), nineteen subjects (10 males and 9 females) was successfully collected to characterize participants including sex, height, age, smoking status, drinking status, duration per defecation, number of defecations per week, the amount of water intake within a day, etc. (Fig 1 and S3 File). As this trial is pair-sample designed, the baseline can be considered as first measured results in each individual. From these subjects, metagenome sequencing was performed based on V3-V4 variable regions of the 16s rRNA in before and after trials, respectively. Sequenced paired-end reads were assembled to generate V3-V4 assembled contigs (S1 Table), and they were successfully annotated as OTUs by QIIME. Average numbers of detected OTUs are 511,879 and 391,516 in before and after trials, respectively (S2 Table). To assess the richness of OTUs, we calculated the *chao1* index and visualized the rarefaction curves (S1 Fig). In the figure, estimated richness of species was converged in before and after trial, but relatively large numbers of OTUs were observed in after 60 days of probiotic administration.

### Measured changes in obesity-related index from the Bioelectrical Impedance Analysis

**Fig 2:**
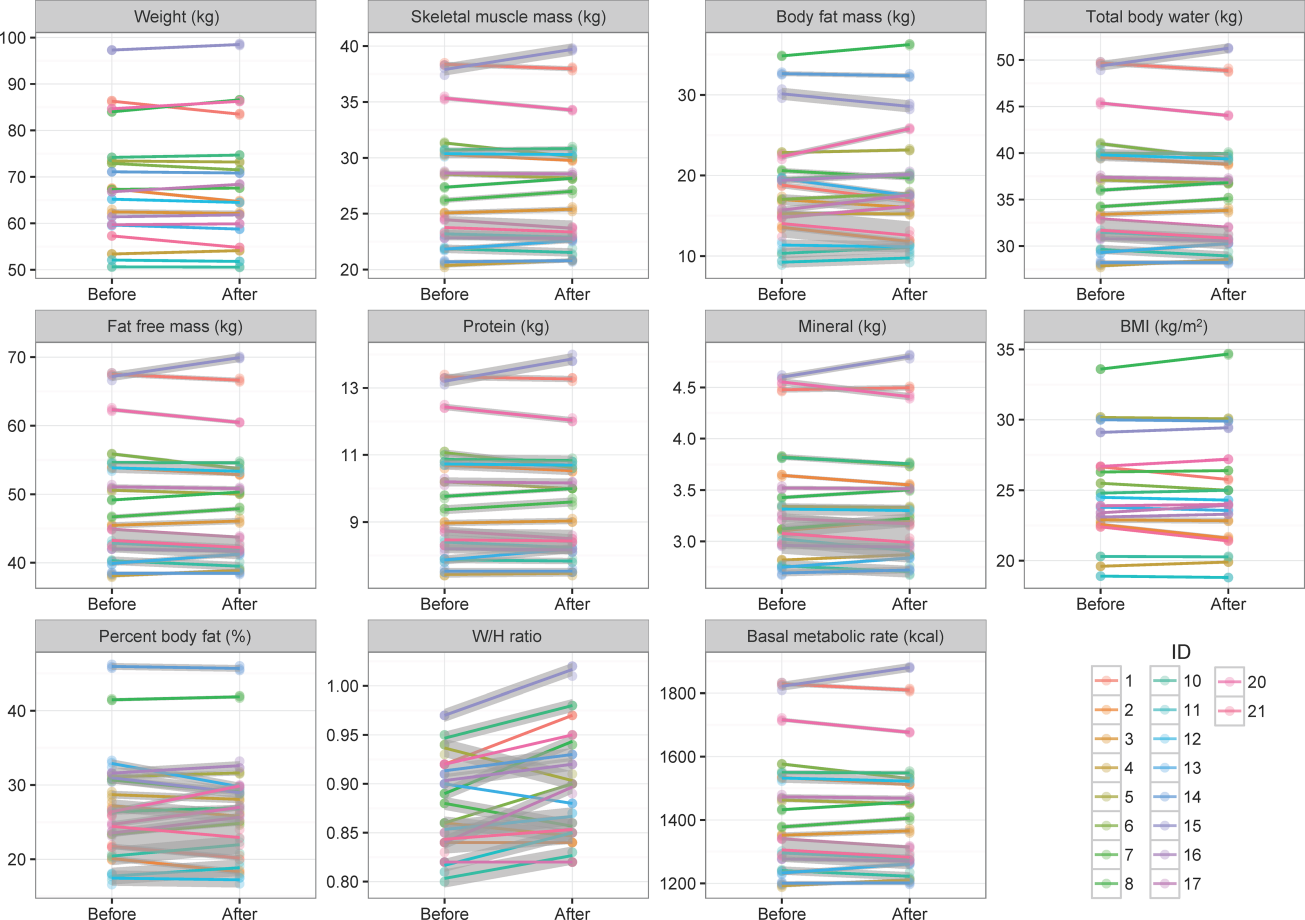
Differences of the results of bioelectrical impedance analysis between before and after 60 days of probiotic administration. A total of 11 indexes were measured by the bioelectrical impedance analyzer. The color of each line represents experimental subjects, and the slope represents the difference between before- and after- trials. The gray shades represent standard errors, which were estimated by three times technical replications. In most cases, measured values are not changed after probiotic administration, but the difference of the W/H ratio was significantly observed in bioelectrical impedance analysis.

The Bioelectrical Impedance Analysis (BIA) was employed to investigate changes in the obesity indexes by 60 days of probiotic administration. The BIA measures were repeatedly measured, three times each for before and after trial, to consider the technical bias of the body composition analysis. In Fig 2, change in trends for 11 body composition related indexes were visualized with their standard error. Most indexes were not changed corresponding to the probiotic intervention, but increasing pattern of the waist to hip ratio (W/H ratio) is shown. To statistically investigate this observation, ANCOVA model was employed with four covariates; height, sex, age, and family information (Table 1). In the results of statistical analysis, significant height effect was observed in skeletal muscle mass, total body water, fat-free mass, protein, mineral, and basal metabolic rate. Also, significant sexual differences were observed in skeletal muscle mass and amount of protein. S2 and S4 Figs provides justifications for these covariates.

**Table 1:** Statistical test results of each trait between before and after trials.

Trait (Response variables)	Height	Sex	Age	Family	Repeat	Stage
**Weight (kg)**	7.53E-02	1.00E+00	1.00E+00	1.00E+00	1.00E+00	1.00E+00
**Skeletal muscle mass (kg)**	2.52E-04*	4.45E-02*	1.00E+00	1.00E+00	9.81E-01	1.00E+00
**Body fat mass (kg)**	1.00E+00	1.00E+00	1.00E+00	5.99E-01	1.00E+00	1.00E+00
**Total body water (kg)**	2.53E-04*	7.00E-02	1.00E+00	1.00E+00	1.00E+00	2.65E-01
**Fat free mass (kg)**	2.42E-04*	7.13E-02	1.00E+00	1.00E+00	1.00E+00	5.85E-01
**Protein (kg)**	2.72E-04*	4.28E-02*	1.00E+00	1.00E+00	1.00E+00	1.00E+00
**Mineral (kg)**	2.74E-04*	6.03E-01	1.00E+00	1.00E+00	1.00E+00	1.00E+00
**BMI** (**kg/m**^**2**^)	1.00E+00	1.00E+00	1.00E+00	1.00E+00	1.00E+00	1.00E+00
**Body fat (%)**	5.51E-01	1.00E+00	1.00E+00	3.93E-01	9.01E-01	1.00E+00
**W/H ratio**	4.32E-01	1.00E+00	1.00E+00	1.00E+00	1.00E+00	8.12E-09*
**Basal metabolic rate (kcal)**	2.42E-04*	7.19E-02	1.00E+00	1.00E+00	1.00E+00	6.15E-01

The value represents Bonferroni’s adjusted P-value and (*) indicates significant result at 5% significance level. The paired repeated analysis-of-covariance (ANCOVA) model was employed. Five explanatory variables were considered in the statistical model such as height, sex, age, technical replications (Repeat), before and after trials (Stage).

Our main interest is to investigate the difference between before and after trials (Fig 2). Therefore, seven-way repeated ANCOVA model was employed including four covariates, technical replication, and individual factor. As a result, only W/H ratio (Bonferroni’s adjusted P-value < 0.05) was significantly changed of the 11 indexes between before and after trials (Table 1) and the others steadily remained.

### Survey analysis to investigate improvement of six gastrointestinal symptoms by probiotic administration

**Fig 3:**
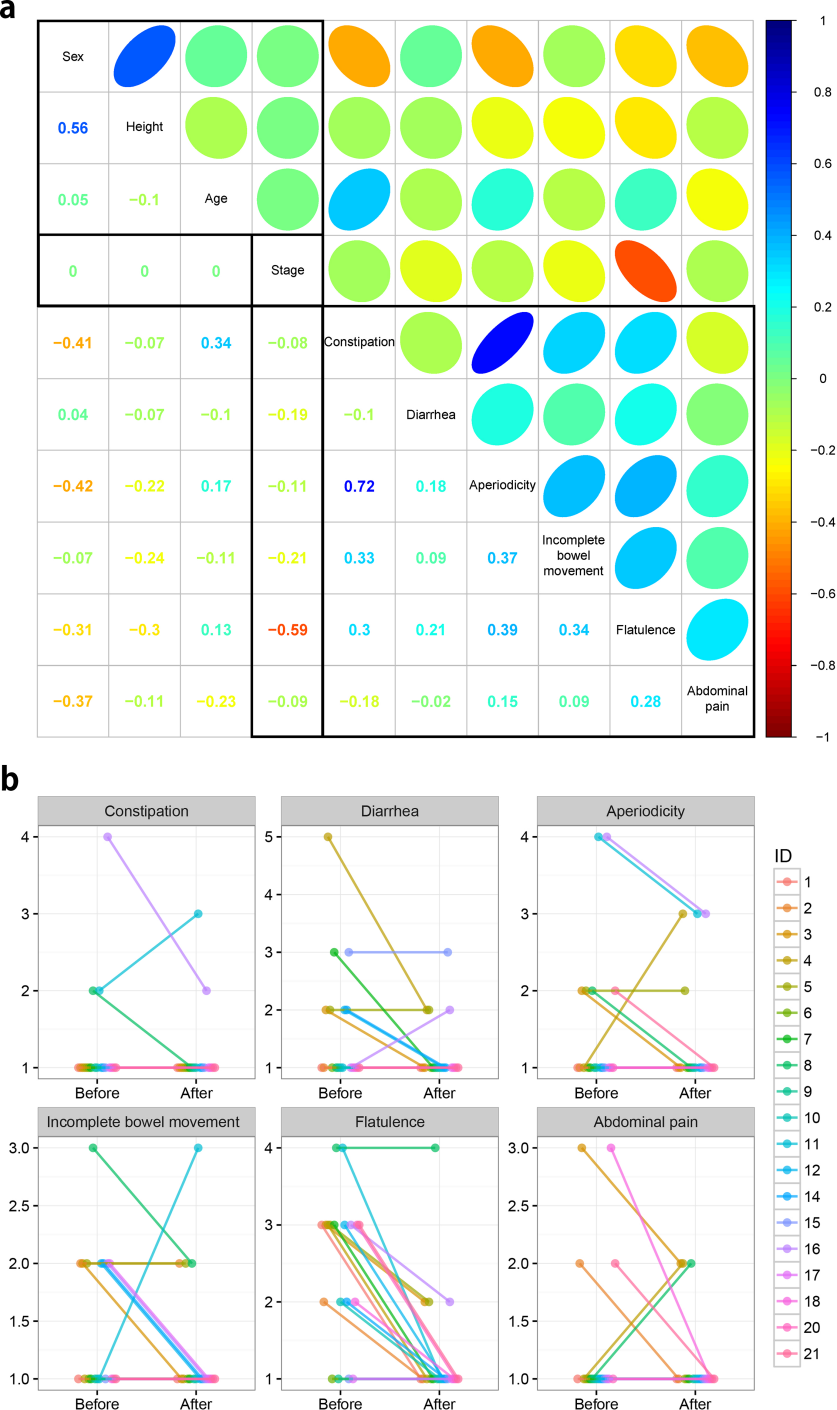
Investigation of relationships between probiotic administration, gastrointestinal symptoms, and covariates. (a) The correlation plot between survey responses on six gastrointestinal symptoms and several covariates. The lower left diagonal represents correlation value and upper right diagonal, the ellipse, represents the strength of the correlation. Two categorical variables; Sex (0: Female and 1: Male) and Stage (0: Before and 1: After), were coded as numerical values to calculate correlation coefficients. (b) Line plots show the responses to the questionnaires on six gastrointestinal symptoms of before- and after- trials.

Six gastrointestinal symptoms were measured by questionnaires in both before and after trials. Correlation coefficients were calculated for simultaneous investigation of the relationship between phenotypes and six gastrointestinal symptoms. In Fig 3A, correlation plot displays linear relationships given various variables. There are three types of correlation structures; phenotype-phenotype, symptoms-symptoms, and phenotype-symptoms relationships. The left-top square represents phenotype-phenotype relationships for the 19 employed samples, and the right-bottom square represents responses of the gastrointestinal symptoms in their survey. Finally, the main relationships of interest, probiotic intervention (Stage) versus the others, were visualized as a rectangle.

In the sample phenotypic relationship, high correlation (0.56) between sex and height was observed. For the six gastrointestinal symptoms, 0.72 positive correlation was observed between constipation and aperiodicity. Only flatulence showed strong negative correlation (-0.59) with probiotic intervention, which means that flatulence symptom was relieved after 60 days of probiotics administration. Except for a small proportion of individuals, most show maintained or alleviated patterns as shown in six gastrointestinal symptoms (Fig 3B). Of these symptoms, strong relief tendency was observed in flatulence. For statistical investigation of symptom’s relief, ordered logistic regression was used, and flatulence was only significantly detected (Bonferroni's adjusted P-value < 0.05) between before and after trials (Table 2).

**Table 2:** Statistical test results of each gastrointestinal symptom between before- and after- trials.

Gastrointestinal symptoms	Height	Sex	Age	Family	Stage
**Constipation**	1.00E+00	9.47E-02	2.14E-01	4.77E-01	1.00E+00
**Diarrhea**	1.00E+00	1.00E+00	1.00E+00	9.85E-01	1.00E+00
**Aperiodicity**	1.00E+00	6.79E-02	1.00E+00	1.00E+00	1.00E+00
**Incomplete bowel movement**	6.07E-01	1.00E+00	1.00E+00	8.29E-01	1.00E+00
**Flatulence**	1.36E-01	5.03E-01	1.00E+00	4.25E-01	2.70E-04*
**Abdominal pain**	1.00E+00	1.11E-01	1.00E+00	1.00E+00	1.00E+00

The value represents Bonferroni’s adjusted P-value and (*) indicates significant result at 5% significance level. For ordinal response variables, ordered logistic regression was employed. Flatulence is the only response that displays significant change between before and after trials.

Another observation is that not only symptom responses were highly correlated between constipation and aperiodicity, but they are also negatively correlated with sex. As shown in S5 Fig, symptom changes of constipation and aperiodicity were observed in female. Such observation also is statistically tested, and sex term was found significant in constipation (Table 2). Except for two gastrointestinal symptoms, tendency changes were observed in other symptoms regardless of sex.

### Metagenome analysis for detecting significantly changed gut-microbiomes’ abundances via probiotic intervention

**Fig 4:**
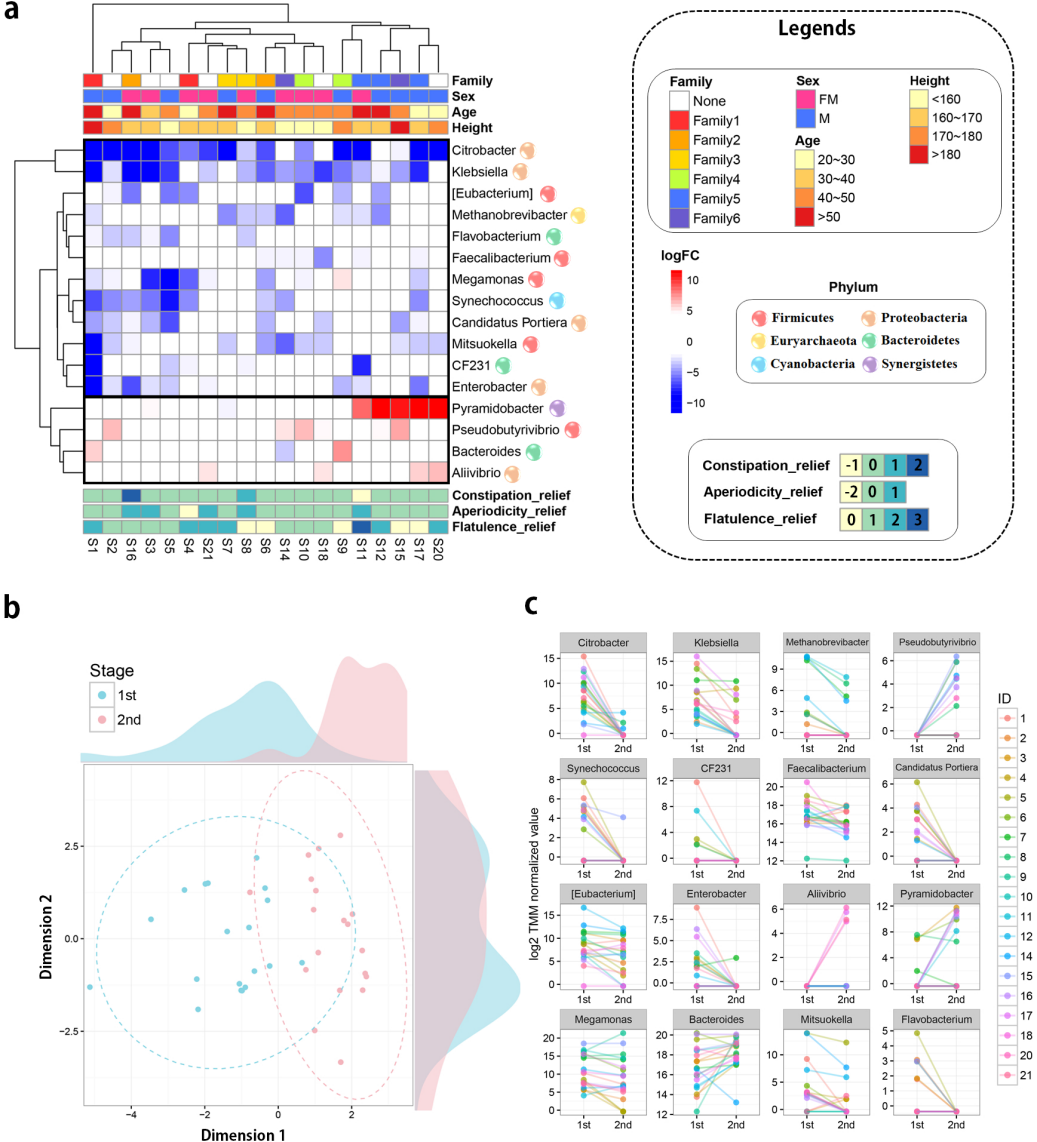
Combined results of the metagenome analysis. (a) Significantly detected genera in the statistical comparison between before and after trials. The ordering for sample and microbiome were determined based on the result of the hierarchical clustering (from far to near ordering). For the clustering analysis, Euclidean distance method was used as distance metric and complete method was employed as breakpoint distance. Total 16 genera were observed at FDR-adjusted P-value < 0.05 and their log2 fold changes were visualized as heat map. The red and blue colors represent up- and down- regulated genera and color intensities show the difference of abundances between before- and after trials. The heat map was annotated by four covariate information and degree of reliefs for three gastrointestinal symptoms. (b) Multidimensional scaling plot (MDS plot) displays gut microbiomes’ change via probiotic intervention. Blue and red colors represent before and after trials, respectively. The dotted circles represent estimated confidence ellipse in multivariate analysis. (c) Line plots of significantly detected 16 genera with their measured abundances. Each line and color display an individual change in OTUs’ abundance. The OTUs’ abundance represents log2 TMM normalized values.

Metagenome analysis was performed to explain phenotypic changes based on the community of gut microbiomes. In metagenome analysis, there are several taxonomic levels from domain to species. Of these taxonomic levels, three most commonly used levels of OTUs (genus, family, and phylum) were used in this study. From the QIIME tool, OTUs’ abundances were quantified in each level, and statistical analysis was performed to detect differentially abundant microbiomes between before and after trials. As a result, 16, 19, and 2 OTUs significantly detected (FDR adjusted P-value < 0.05) in the genus, family, and phylum taxonomic level, respectively (S3 and S5 Tables). Of these results, genus level results were primarily focused on, because it is a more specific level compared to the others. Differences of 16 significantly detected 16 genera were visualized as a heat map with diverse phenotypic information; family, sex, age, height, and three interesting survey results (Fig 4A). In addition, hierarchical clustering analysis was performed among OTU abundances of the individuals. When the clustering and annotated results are combined, no clear explanation could be made, but clusters were partly ordered by family information, which means that community of the gut microbiota is heterogeneous. From the clustering result, Sample ID_1 (S1) individual was observed as an out-group sample in the clustering analysis. The diversity plot explained the reason that his community pattern is distinct from other samples (S6 Fig). In this figure, microbiome diversity of the S1 sample was extremely reduced after 60 days of probiotic intervention. Such observation could be highly affected by the difference in reads’ production, generated numbers of reads, and detected numbers of OTUs that were investigated (S1 and S2 Tables). The numbers of reads and OTUs are found to increase in after administration, while the diversity of microbiota extremely decreased. Furthermore, the degree of diversity alteration by the probiotic administration is correlated by sex (-0.6), height (-0.59), and age (-0.47) (S7 Fig). These results suggest that degree of diversity change highly depends on sex, height, and age. Such observation is due to S1 out group individual, who is characterized as senior, tall, and male.

Finally, of significantly detected 16 OTUs, 12 and 4 genera were respectively down and up-regulated after 60 days of probiotic intervention. To visually illustrate the statistical results, multidimensional scaling (MDS) analysis was performed with abundances of the16 significant genera. As a result, a clear pattern was observed between before and after trials (Fig 4B). In the family taxonomic level, 19 samples were separated corresponding to the stage (S8 and S9 Figs). Finally, two phyla OTUs, *Euryachaeota* and *SAR406*, were detected significant (S10 Fig). In Fig 4C, these OTUs were visualized as line-plots, ordered by their probability values. Of these OTUs, top 3 OTUs; *Citrobacter* (-9.22), *Klebsiella* (-5.49), and *Methanobrevibacter* (-3.88), were reduced significantly after probiotic administration in terms of log2 fold-change (logFC).

### Network analysis reveals that flatulence is highly affected by probiotic intervention and *Methanobrevibacter*’s abundance

**Fig 5:**
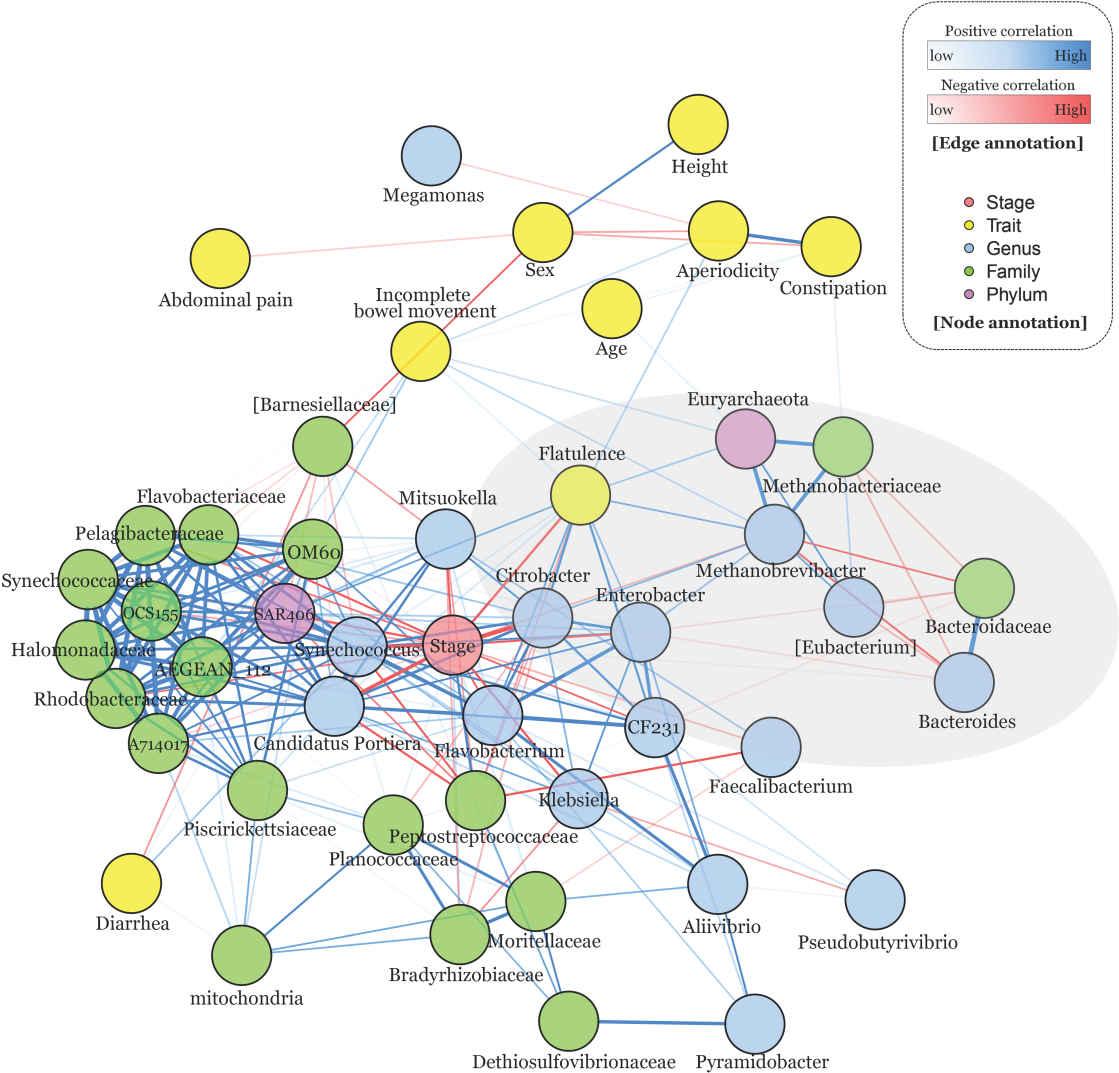
The result of correlation-based network analysis. Significantly detected OTUs (FDR adjusted P-value < 0.05) in the genus, family, and phylum taxonomic levels. The network plot is drawn with phenotypes and six gastrointestinal symptoms. Spearman’s correlation test was employed, and only the significantly detected relationships (FDR adjusted P-value < 0.05) were visualized in the network plot. The edges show the strength of the correlation relationships and their colors represent positive and negative correlation as blue and red colors, respectively. The gray ellipse represents significantly observed traits after 60 days of probiotic intervention and their correlated OTUs (S11 Fig).

Investigation for phenotypic alteration was performed in the bioelectrical impedance and questionnaires survey analysis. Furthermore, metagenome analysis was performed on the significantly abundant microbiomes. Although in previous studies, the phenotypic and metagenomic changes in response to the probiotic administration have been independently investigated, yet the relationships between metagenomic change and phenotypic alteration have never been simultaneously identified. In addition, the multi-level taxonomic structure was not considered in the analysis. Correlation based network analysis was performed with survey results for six gastrointestinal symptoms, phenotypes, and significantly detected OTUs in the genus, family, and phylum levels, for simultaneous investigation. There were several significant relationships (FDR adjusted P-value < 0.05), and those complex relationships were visualized in network plot (Fig 5). As most observed OTUs were down-regulated by probiotic administration (Figs 4A and 3C), the network plot displays several negative correlated OTUs with probiotic intervention node (Stage) in the core. Of several trait-nodes, only flatulence was significantly correlated with the stage node, which is a thread of connection with the result of survey analysis (Fig 3 and Table 2). To further explore relationships, flatulence-microbiome relationships were focused on (shades of gray in Fig 5 and S11 Fig). As a result, the flatulence is positively correlated (Spearman correlation: 0.45) with the *matanobrevibacter* (Genus) and *Euyarchaeota* (Phylum). In addition, *Methanobacteriaceae* is also positively correlated with tho two nodes, and they establish a triangular relationship. As *matanobrevibacter* is included in the *Methanobacteriaceae* family of *Euyarchaeota* phylum, those 1:1:1 multi taxonomic relationship lends support to the identified relationship. *Methanobrevibacter* was negatively correlated (-0.54) with *bacteroides* (and *bacteroidaceae* [Family]).

## Discussion

Through metagenome analysis on 16S rRNA taxonomic data, we present that probiotic intervention reduced the abundance of potential bacteria such as *Citrobacter* and *Klebsiella* in the human gut microbial community (Figs 4A and 3C). *Citrobacter* (-9.22 fold reduced in 60 days of probiotic intervention, S3 Table) is a gram-negative coliform bacterium that is affiliated with *Enterobacteriaceae* and an enteric pathobiont, which occasionally provoke the urinary tract infection, meningitis, and sepsis. Of note, *Citrobacter rodentium*, a pathogen in mice, have been studied to elucidate the mechanism of enteric pathogenesis by commensal bacteria [29]. Furthermore, previous mouse studies have indicated that probiotics attenuated the enteric infection of *Citrobacter rodentium* [30, 31]. In the case of *Klebsiella* (-5.49 fold attenuated in probiotic intervention, S3 Table), the genus is also a gram-negative bacteria affiliated with *Enterobacteriaceae* and can cause pneumonia and various diseases such as urinary tract infection, septicemia, etc. The infection study of *Klebsiella pneumoniae* suggests that administration of *Bifidobacterium longum* prevented mice from pneumoniae-induced death via immunomodulatory effect [32].

*Methanobrevibacter*, a genus of archaea (3.87 fold reduced after probiotic administration, S3 Table), converts hydrogen gas to methane. Elimination of hydrogen enhances the efficiency of microbial fermentation for carbohydrate substrates in the human gut ecosystem [33]. In the human digestive tract, *Methanobrevibacter smithii* is the most dominant methanogen constituting 94% of the methane-producing microbial population [34]. Studies using artificially colonized mice demonstrated that *Methanobrevibacter smithii* influences host energy harvest and obesity via enhancing gut microbiota to digest dietary polysaccharides [35]. *Methanobrevibacter* had been recognized as a methanogenic archaea that do not cause diseases in human except for flatulence or uncomfortable gas evacuation. However, recent metagenomics studies suggest otherwise; methane production of *Methanobrevibacter* may have relationships with the pathogenesis of digestive tract symptoms and diseases in human [33, 36]. Our result also reveals that relative abundances of the *Methanobrevibacter* were highly correlated with the degree of flatulence (Fig 3 and Table 2). Furthermore, interaction effect between such relationship and probiotic intervention was newly introduced in this study (Fig 5 and S11 Fig). In reality, it is not obvious whether the reduction of flatulence resulted from lower methane production or lower microbial fermentation due to the lower elimination of hydrogen gas, but *Methanobrevibacter* would be a strong candidate to alleviate flatulence. Although laxatives or enema treatment has shown to reduce methane production [37], no clinical trials and drugs have been developed to manipulate *Methanobrevibacter smithii*, a major methane producer in human. In this respect, although further study is required to clarify causal relationship between *Methanobrevibacter* and pathogenesis of various diseases in human, our finding would be helpful to develop drugs for the bowel disease. As for the limitation of this study, our primary focus was on the microbial community change between pre- and post-intervention, and for this reason, the placebo group was not considered in this study. With this in mind, a further study that considers placebo effect is required to investigate the confounding effect between probiotic intervention and flatulence.

As a human feces’ metagenome study, it is practically impossible to control whole experimental variables (i.e. personal drug use). Recently, researchers have continuously attempted to measure the phenotypic variation of the microbiome community in large population data, the importance of the covariate adjustment is elucidated for accurate estimation of statistical model on highly heterogeneous data [38]. In this study, although we attempted to consider as many covariates as possible, highly heterogeneous patterns make it more difficult to identify differences between trials (Figs 4A and 3C). This result would mean the importance of personalized probiotics administration. One of the examples could be found in both Fig 2 and S5 Fig. Of the six gastrointestinal symptoms, we observed two, constipation and aperiodicity, to show sex-dependent symptom relief; only females show relief of symptoms after 60 days of probiotic intervention. Another evidence of the necessity for personalized probiotics administration could be found in Fig 3 and S6 Fig. In this figure, only S1 sample shows distinct pattern compared to others in terms of microbiome diversity. We believe the extreme reduction of microbiome diversity and abundance, of S1 individual, resulted from the medical use of antibiotics towards the end of probiotic intervention period. Antibiotics have shown certain limitation to suppress the abundance of human commensal *Methanobrevibacter*, which is resistant to the majority of antibiotics [39]. The use of antibiotics also leads to extreme disturbance in the human gut ecosystem that may provoke other detrimental consequences in gut health [40]. Also, the antibiotic administration induces acute disturbance throughout the intestinal microbiota losing diversity and abundance [14, 40]. Given this information, the development of personalized probiotics should measure their effects not only under changeable host conditions but also on diverse gut microbiome communities. Although our study employed a small number of samples, we expect that the explanation of such complex relationships, the host-microbe-probiotics interaction, will be materialized in the near future with large enough samples.

In the 21^st^ century, there is a growing interest in marker detection related to obesity and malnutrition. While numerous researchers detected several candidate markers in diverse biological datasets (i.e. genetic data, transcriptome data, etc.), they have failed to find distinctive evidence for those markers because of complexity in obesity-related traits [41–43]. Especially, the missing heritability problem has been a recurrent issue in obesity research, and several studies tried to tie gut microbiota with the genetic information to improve in terms of explained variance. As a result, several candidates were suggested in an effort to identify causal microbiome related to obesity in diverse species and experimental designs [44, 45]. The majority of previous researches attempted to determine the relationship between probiotics and obesity [7, 46–48]. Unfortunately, no known method can clearly identify the causal relationship between obesity and probiotic intervention (although some *Lactobacillus* species were detected as candidate markers), and most researchers emphasize further study be required due to metabolic complexity and sample specificity (i.e. host-specific pattern). However, we found that W/H ratio increased significantly by probiotic intervention; the result opposes our expectation (Fig 2 and Table 1). We suspect that such result derived from two reasons. First, W/H ratio was measured based on the bioelectrical impedance analysis, which embeds high technical bias. As shown in Fig 2, each trial was measured three times for estimation of technical variability, which is visualized with a gray shade. Some traits, such as weight, skeletal muscle mass, and body fat mass, can be relatively measured with low technical variance by the bioelectrical impedance machine. In contrast, there is high technical variance in W/H ratio, mineral, and body fat percent, and which leads to a deviation from accurate estimation using bioelectrical impedance machine only. The second reason is that there could be the seasonal effect for the second measurement (after 60 days of probiotic intervention). The period was overlapped with year-end parties. In Korean society, an individual attends one or more year-end parties that are either thrown at a personal level (i.e. friends and families) or company level (i.e. workplace dinner party). Since high consumption of meat and alcohol is usual, such custom would induce the W/H ratio increment. Unfortunately, it is practically impossible to consider such unmeasured environmental effects in our study. Therefore, a further study should be conducted with large sample size and controlled diet. Based on the observations, we conclude that although W/H ratio displays statistically significant change, there is no direct link between obesity-related traits and probiotic intervention, which is concordant with previous reports.

Conclusively, our analyses provide a blueprint for host-probiotics, microbe-probiotics, and host-microbe-probiotics interaction in the human intestinal ecosystem. The results highlight probiotic intervention may reduce the flatulence through downregulation of *Methanobrevibacter* abundance. Further research and application of probiotics targeting *Methanobrevibacter* may contribute to the alleviation of gastrointestinal symptoms and diseases in human.

## Supporting information

S1 Table Number of successfully assembled sequences. (PDF)

S2 Table Observed OTUs’ abundances in each sample. (PDF)

S3 Table The 16 significantly detected genera (FDR adjusted P-value < 0.05). (PDF)

S4 Table The 19 significantly detected family-level OTUs (FDR adjusted P-value < 0.05). (PDF)

S5 Table The two significantly detected phyla (FDR adjusted P-value < 0.05). (PDF)

S1 Fig Rarefaction curves to investigate OTUs were measured by increasing number of sequences. (a) before- and (b) after- metagenome experiments. In the plot, chao1 index was employed to measure alpha diversity in observed OTUs.
(PDF)

S2 Fig Differences in the results of bioelectrical impedance analysis between before and after 60 days of probiotic administration (Line color represents sex, female [FM] and male [M]). (PDF)

S3 Fig Differences in the results of bioelectrical impedance analysis between before and after 60 days of probiotic administration (Line color represents age). (PDF)

S4 Fig Differences in the results of bioelectrical impedance analysis between before and after 60 days of probiotic administration (Line color represents height). (PDF)

S5 Fig Investigation of interaction effects between sex and relief of gastrointestinal symptoms. (PDF)

S6 Fig Comparing chao1 indexes between before and after trials to investigate microbiome’s diversity change. (PDF)

S7 Fig Correlation plot to investigate relationships between microbiome diversity and others. (PDF)

S8 Fig MDS plot with significantly detected 19 OTUs in family taxonomic level. (PDF)

S9 Fig Line-plots of the 19 significantly detected family-level OTUs. (PDF)

S10 Fig The two significantly detected phyla between before- and after- trials. (PDF)

S11 Fig Correlation plot for significantly detected traits and related OTUs. (PDF)

S1 File Trial protocol. (PDF)

S2 File TREND form. (PDF)

S3 File Participant information. (XLSX)

S4 File CONSORT form. (PDF)
