# Supplementary material for: Methanobrevibacter attenuation via probiotic intervention reduces flatulence in adult human: A non-randomised paired-design clinical trial of efficacy
Source: PLoS One. 2017 Sep 22;12(9):e0184547. doi: 10.1371/journal.pone.0184547 (PMC5609747; doi:10.1371/journal.pone.0184547)
Supplement: S1 Table — (PDF) [file pone.0184547.s001.pdf]

S1 Table. Number of successfully assembled sequences

ID	Total # of pairs	Combined_ Pairs	Percent combined	Total # of pairs	Combined_ Pairs	Percent combined
1	659,608	436,804	66.22%	666,744	485,977	72.89%
2	535,965	357,966	66.79%	807,347	601,097	74.45%
3	961,467	614,881	63.95%	660,574	507,442	76.82%
4	701,337	438,468	62.52%	740,494	498,639	67.34%
5	640,851	435,490	67.95%	871,912	663,811	76.13%
6	717,033	486,245	67.81%	334,602	255,793	76.45%
7	862,555	567,368	65.78%	700,470	530,556	75.74%
8	586,821	375,763	64.03%	790,058	582,793	73.77%
9	808,002	549,937	68.06%	726,022	504,805	69.53%
10	885,158	579,954	65.52%	584,101	427,670	73.22%
11	697,989	480,262	68.81%	258,279	190,291	73.68%
12	1,559,696	1,078,672	69.16%	569,246	440,592	77.40%
14	937,413	653,688	69.73%	598,578	457,070	76.36%
15	728,084	475,277	65.28%	391,549	289,772	74.01%
16	706,993	506,800	71.68%	639,145	475,502	74.40%
17	739,033	520,020	70.36%	351,069	264,555	75.36%
18	1,109,510	597,002	53.81%	525,082	389,329	74.15%
20	632,610	425,657	67.29%	366,555	277,983	75.84%
21	1,014,321	755,294	74.46%	675,617	350,901	51.94%
	Before trial			After trial		
