# Supplementary material for: Methanobrevibacter attenuation via probiotic intervention reduces flatulence in adult human: A non-randomised paired-design clinical trial of efficacy
Source: PLoS One. 2017 Sep 22;12(9):e0184547. doi: 10.1371/journal.pone.0184547 (PMC5609747; doi:10.1371/journal.pone.0184547)
Supplement: S2 Table — (PDF) [file pone.0184547.s002.pdf]

**S2 Table. Observed OTUs' abundances in each sample**

| ID | Before | After | Before-After |
|---|---|---|---|
| **1** | 403,514 | 441,949 | -38,435 |
| **2** | 336,086 | 548,542 | -212,456 |
| **3** | 583,223 | 465,055 | 118,168 |
| **4** | 411,158 | 437,118 | -25,960 |
| **5** | 412,500 | 609,146 | -196,646 |
| **6** | 453,077 | 236,030 | 217,047 |
| **7** | 518,747 | 473,792 | 44,955 |
| **8** | 350,965 | 521,882 | -170,917 |
| **9** | 518,040 | 461,093 | 56,947 |
| **10** | 550,258 | 390,568 | 159,690 |
| **11** | 451,234 | 176,054 | 275,180 |
| **12** | 1,024,835 | 412,634 | 612,201 |
| **14** | 620,032 | 414,397 | 205,635 |
| **15** | 452,960 | 268,565 | 184,395 |
| **16** | 474,057 | 430,098 | 43,959 |
| **17** | 492,563 | 246,498 | 246,065 |
| **18** | 553,490 | 348,439 | 205,051 |
| **20** | 405,017 | 257,754 | 147,263 |
| **21** | 713,954 | 299,192 | 414,762 |
