# Supplementary material for: Methanobrevibacter attenuation via probiotic intervention reduces flatulence in adult human: A non-randomised paired-design clinical trial of efficacy
Source: PLoS One. 2017 Sep 22;12(9):e0184547. doi: 10.1371/journal.pone.0184547 (PMC5609747; doi:10.1371/journal.pone.0184547)
Supplement: S3 Table — (PDF) [file pone.0184547.s003.pdf]

S3 Table. The 16 significantly detected genera (FDR adjusted P-value < 0.05)

Phylum	Family	Genus	P_value	FDR	logFC
Bacteroidetes	[Paraprevotellaceae]	**CF231**	0.000138	2.84E-03	-2.96525
Bacteroidetes	Bacteroidaceae	**Bacteroides**	0.004042	3.65E-02	1.151583
Bacteroidetes	Flavobacteriaceae	**Flavobacterium**	0.006028	4.82E-02	-2.39396
Cyanobacteria	Synechococcaceae	**Synechococcus**	3.11E-05	7.95E-04	-4.64196
Euryarchaeota	Methanobacteriaceae	**Methanobrevibacter**	9.94E-06	3.31E-04	-3.87502
Firmicutes	Lachnospiraceae	**Pseudobutyrivibrio**	1.03E-05	3.31E-04	4.216203
Firmicutes	Ruminococcaceae	**Faecalibacterium**	0.00016	2.84E-03	-1.13834
Firmicutes	Erysipelotrichaceae	**[Eubacterium]**	0.001157	1.65E-02	-2.05939
Firmicutes	Veillonellaceae	**Megamonas**	0.002199	2.17E-02	-1.9666
Firmicutes	Veillonellaceae	**Mitsuokella**	0.004282	3.65E-02	-2.63018
Proteobacteria	Enterobacteriaceae	**Citrobacter**	2.44E-11	3.13E-09	-9.2212
Proteobacteria	Enterobacteriaceae	**Klebsiella**	6.26E-09	4.01E-07	-5.49062
Proteobacteria	Halomonadaceae	**Candidatus Portiera**	0.000177	2.84E-03	-3.57809
Proteobacteria	Enterobacteriaceae	**Enterobacter**	0.00147	1.83E-02	-3.41043
Proteobacteria	Vibrionaceae	**Aliivibrio**	0.001576	1.83E-02	2.468071
Synergistetes	Dethiosulfovibrionaceae	**Pyramidobacter**	0.001797	1.92E-02	3.771663
