# Supplementary material for: Methanobrevibacter attenuation via probiotic intervention reduces flatulence in adult human: A non-randomised paired-design clinical trial of efficacy
Source: PLoS One. 2017 Sep 22;12(9):e0184547. doi: 10.1371/journal.pone.0184547 (PMC5609747; doi:10.1371/journal.pone.0184547)
Supplement: S4 Table — (PDF) [file pone.0184547.s004.pdf]

S1 Table. The 19 significantly detected family-level OTUs (FDR adjusted P-value < 0.05)

Phylum	Family	P_value	FDR	logFC
Actinobacteria	**OCS155**	1.39E-08	8.81E-07	-4.44248
Firmicutes	**Peptostreptococcaceae**	2.07E-08	8.81E-07	4.877239
Proteobacteria	**Pelagibacteraceae**	1.77E-05	3.76E-04	-4.94507
Proteobacteria	**Halomonadaceae**	1.33E-05	3.76E-04	-3.85315
Euryarchaeota	**Methanobacteriaceae**	3.01E-05	5.12E-04	-3.75728
SAR406	**A714017**	8.59E-05	1.22E-03	-3.2943
Cyanobacteria	**Synechococcaceae**	0.000222	2.70E-03	-4.36532
Bacteroidetes	**Flavobacteriaceae**	0.000279	2.96E-03	-3.87584
Proteobacteria	**Rhodobacteraceae**	0.00041	3.16E-03	-3.65558
Proteobacteria	**AEGEAN_112**	0.000372	3.16E-03	-2.99686
Proteobacteria	**OM60**	0.00034	3.16E-03	-2.9729
Proteobacteria	**Piscirickettsiaceae**	0.000626	4.43E-03	-2.76107
Synergistetes	**Dethiosulfovibrionaceae**	0.001699	1.11E-02	3.665901
Bacteroidetes	**[Barnesiellaceae]**	0.003051	1.73E-02	2.113337
Firmicutes	**Planococcaceae**	0.003037	1.73E-02	2.369546
Proteobacteria	**Moritellaceae**	0.005573	2.96E-02	1.985535
Proteobacteria	**Bradyrhizobiaceae**	0.006686	3.34E-02	2.047661
Bacteroidetes	**Bacteroidaceae**	0.010298	4.64E-02	1.065054
Proteobacteria	**mitochondria**	0.010364	4.64E-02	-1.97439
