# Supplementary material for: Methanobrevibacter attenuation via probiotic intervention reduces flatulence in adult human: A non-randomised paired-design clinical trial of efficacy
Source: PLoS One. 2017 Sep 22;12(9):e0184547. doi: 10.1371/journal.pone.0184547 (PMC5609747; doi:10.1371/journal.pone.0184547)
Supplement: S5 Table — (PDF) [file pone.0184547.s005.pdf]

S1 Table. The two significantly detected phyla (FDR adjusted P-value < 0.05)

Domain	Phylum	P_value	FDR	logFC
Archaea	**Euryarchaeota**	8.86E-06	1.51E-04	-3.93771
Bacteria	**SAR406**	0.000339	2.88E-03	-3.16416
