# Supplementary material for: Methanobrevibacter attenuation via probiotic intervention reduces flatulence in adult human: A non-randomised paired-design clinical trial of efficacy
Source: PLoS One. 2017 Sep 22;12(9):e0184547. doi: 10.1371/journal.pone.0184547 (PMC5609747; doi:10.1371/journal.pone.0184547)
Supplement: S1 Fig — (a) before- and (b) after- metagenome experiments. In the plot, chao1 index was employed to measure alpha diversity in observed OTUs. (PDF) [file pone.0184547.s006.pdf]

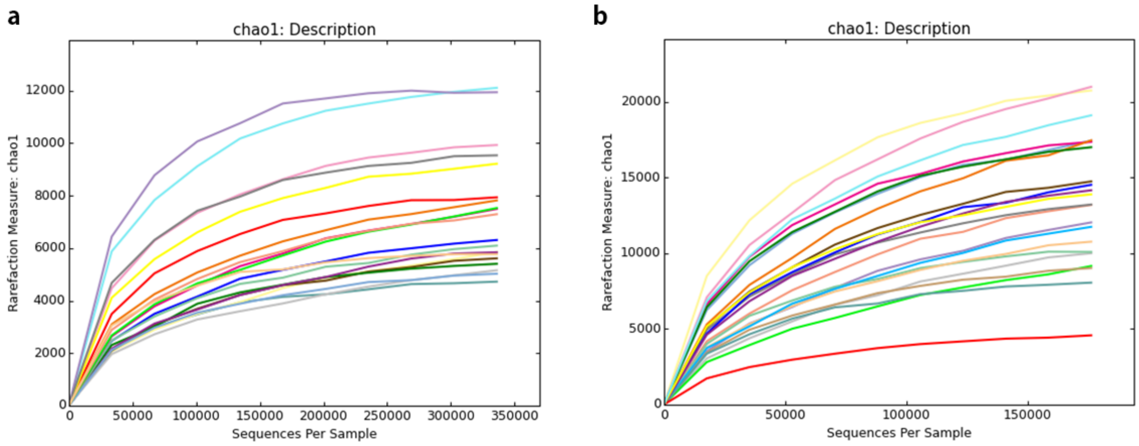

S1 Fig. Rarefaction curves to investigate OTUs were measured by increasing number of sequences in (a) before- and (b) after- metagenome experiments. In the plot, *chao1* index was employed to measure alpha diversity in observed OTUs.
