# Supplementary material for: Methanobrevibacter attenuation via probiotic intervention reduces flatulence in adult human: A non-randomised paired-design clinical trial of efficacy
Source: PLoS One. 2017 Sep 22;12(9):e0184547. doi: 10.1371/journal.pone.0184547 (PMC5609747; doi:10.1371/journal.pone.0184547)
Supplement: S5 Fig — (PDF) [file pone.0184547.s010.pdf]

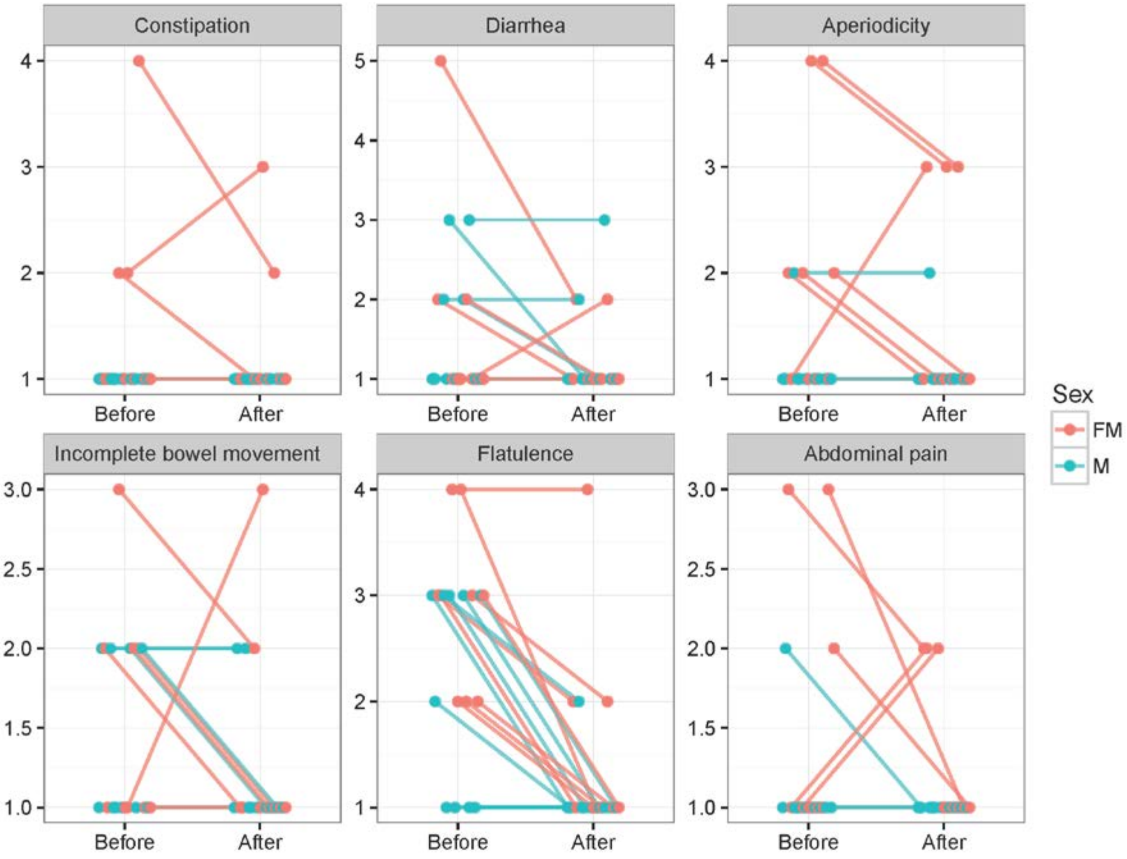

S5 Fig. Investigation of interaction effects between sex and relief of gastrointestinal symptoms
