# Supplementary material for: Methanobrevibacter attenuation via probiotic intervention reduces flatulence in adult human: A non-randomised paired-design clinical trial of efficacy
Source: PLoS One. 2017 Sep 22;12(9):e0184547. doi: 10.1371/journal.pone.0184547 (PMC5609747; doi:10.1371/journal.pone.0184547)
Supplement: S6 Fig — (PDF) [file pone.0184547.s011.pdf]

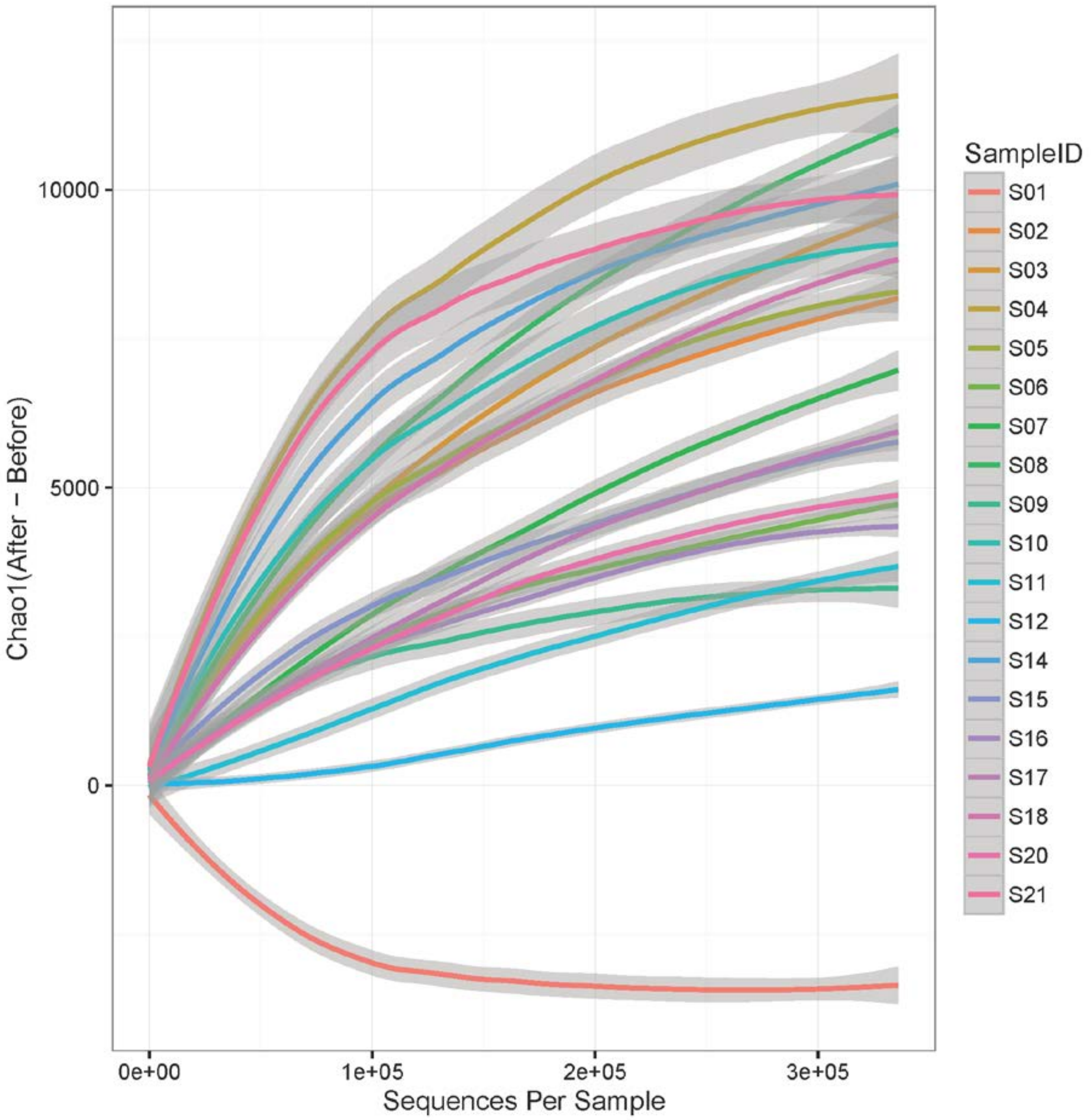

S6 Fig. Comparing chao1 indexes between before and after trials to investigate microbiome's diversity change.

Y-axis is the difference between before and after trials and X-axis is the number of sequences per sample. The result shows that diversity of S1 sample is only reduced.
