# Supplementary figures and images for: Methanobrevibacter attenuation via probiotic intervention reduces flatulence in adult human: A non-randomised paired-design clinical trial of efficacy
Source: PLoS One. 2017 Sep 22;12(9):e0184547. doi: 10.1371/journal.pone.0184547 (PMC5609747; doi:10.1371/journal.pone.0184547)

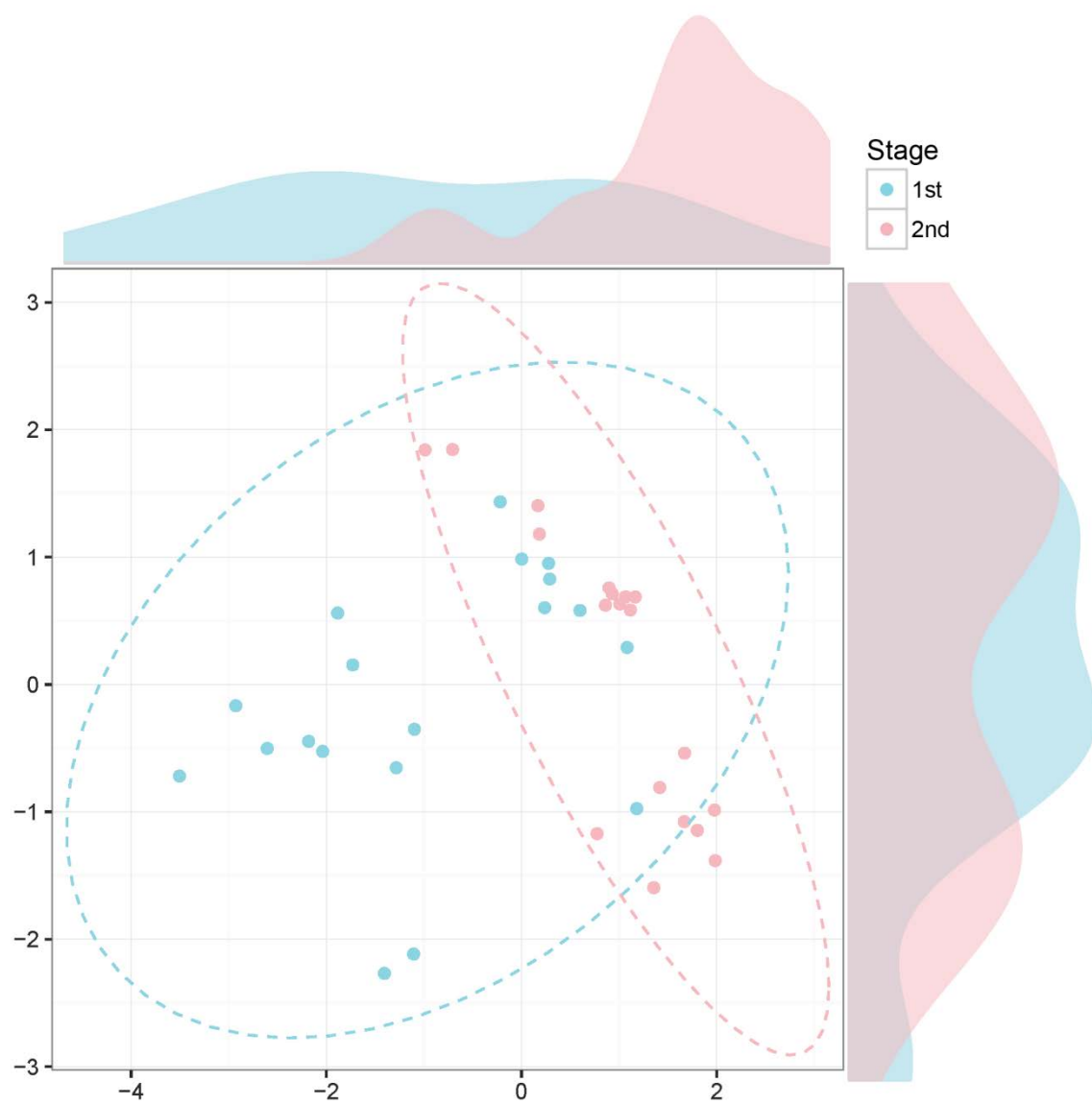

**S1 Fig.** MDS plot with significantly detected 19 OTUs in family taxonomic level

Supplement: S8 Fig — (PDF) [file pone.0184547.s013.pdf]

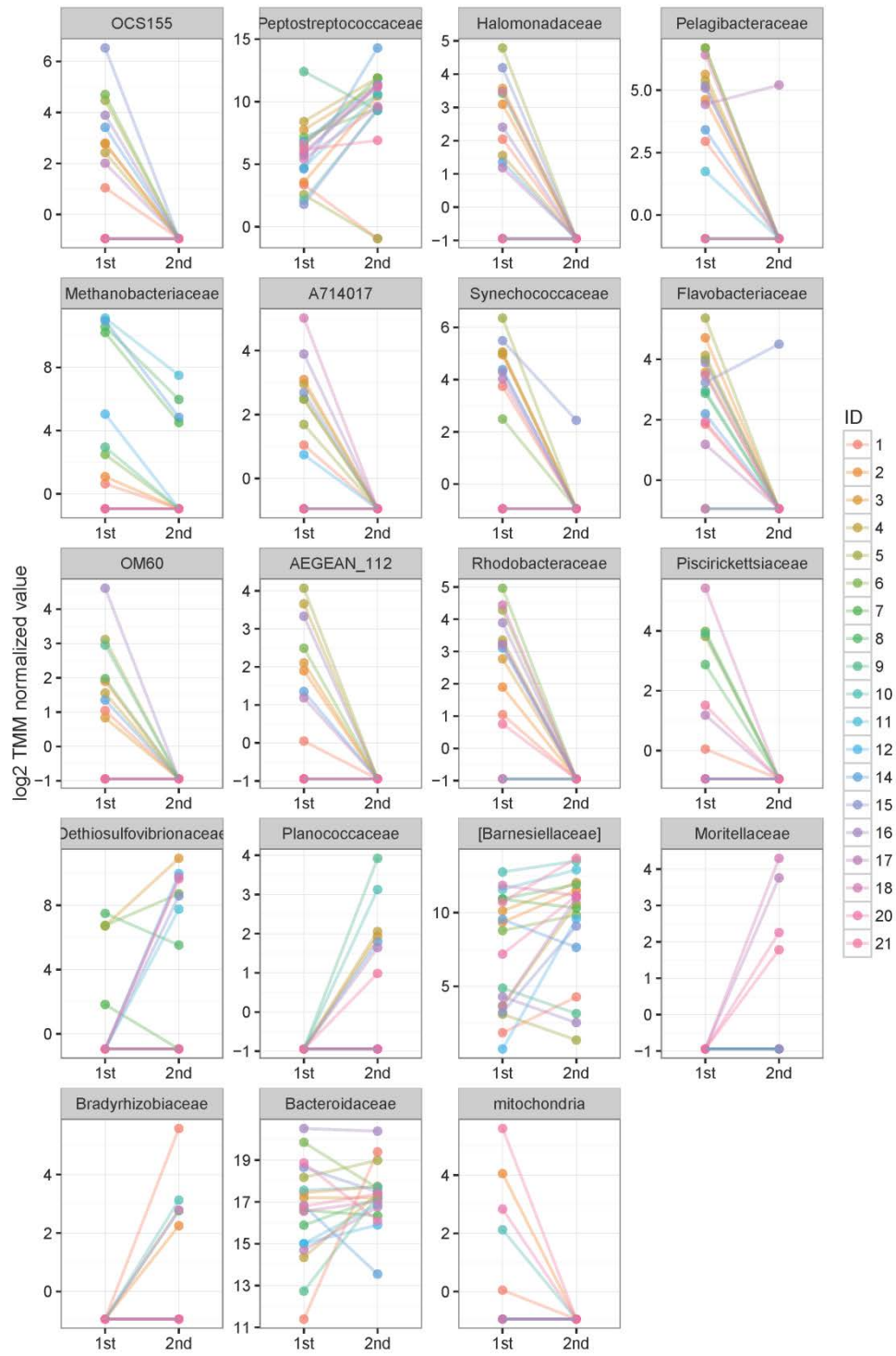

**S9 Fig. Line-plots of the 19 significantly detected family-level OTUs.**

Supplement: S9 Fig — (PDF) [file pone.0184547.s014.pdf]

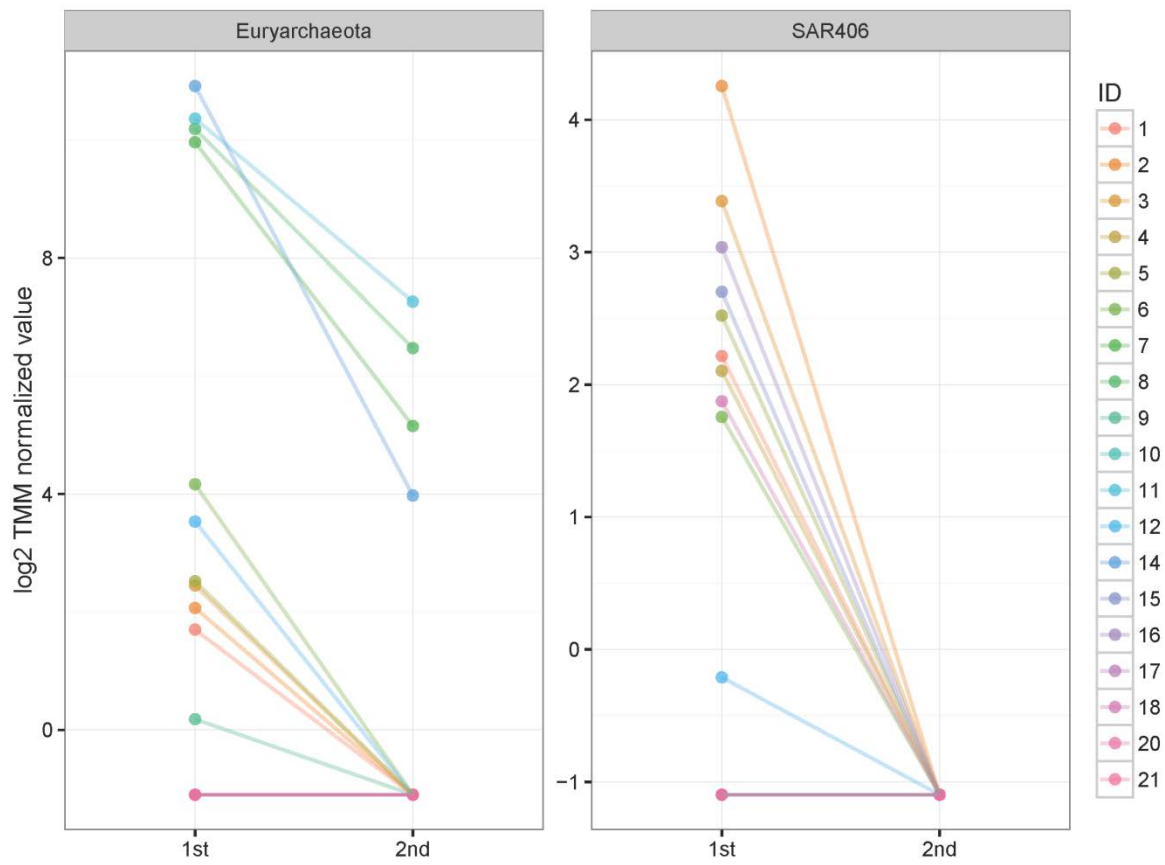

**S10 Fig. The two significantly detected phyla between before- and after- trials.**

Supplement: S10 Fig — (PDF) [file pone.0184547.s015.pdf]

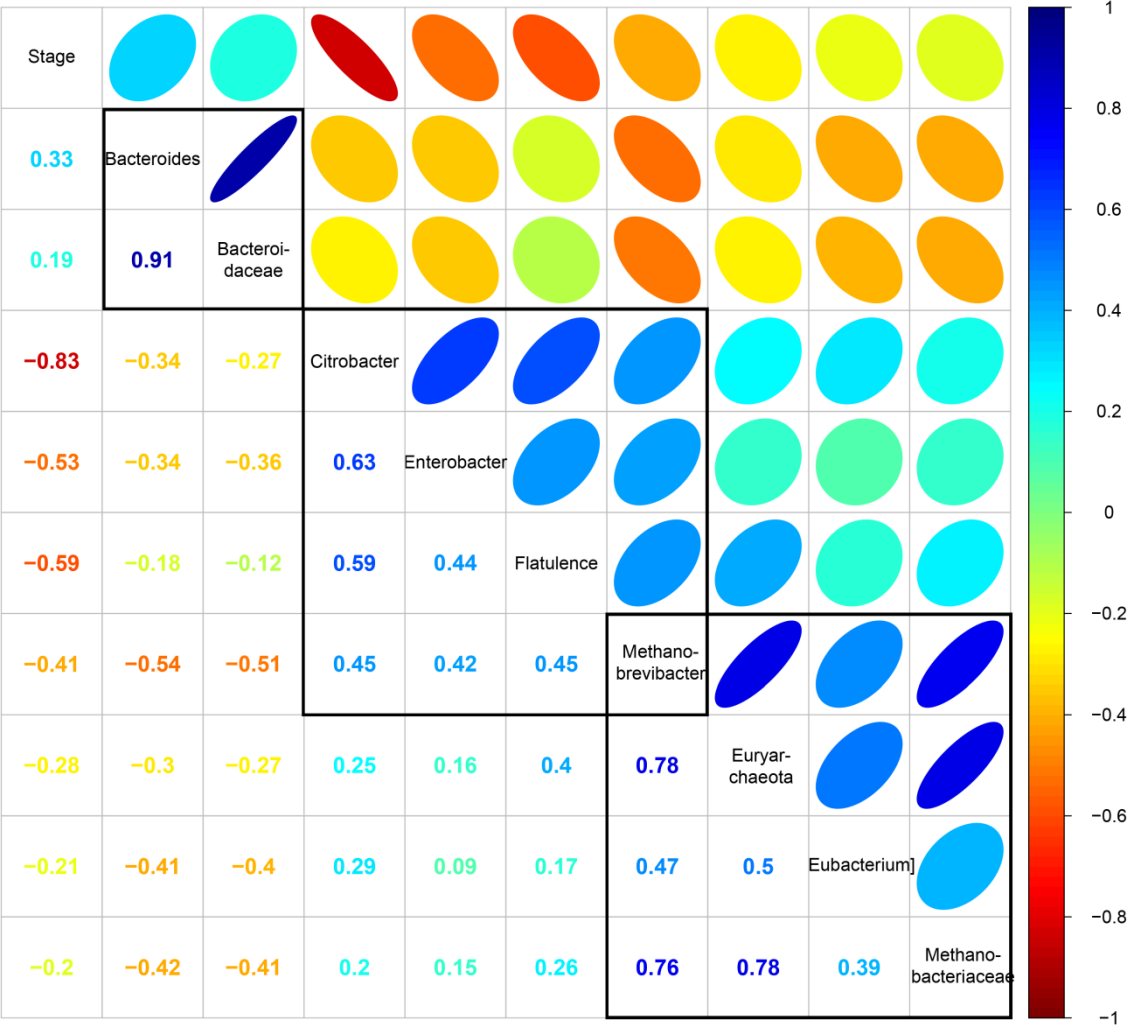

S11 Fig. Correlation plot for significantly detected traits and related OTUs.

Supplement: S11 Fig — (PDF) [file pone.0184547.s016.pdf]
