# Supplementary material for: Methanobrevibacter attenuation via probiotic intervention reduces flatulence in adult human: A non-randomised paired-design clinical trial of efficacy
Source: PLoS One. 2017 Sep 22;12(9):e0184547. doi: 10.1371/journal.pone.0184547 (PMC5609747; doi:10.1371/journal.pone.0184547)
Supplement: S1 File — (PDF) [file pone.0184547.s017.pdf]

Trial protocol
(Phase I study)

Scientific Title: Identification of gut microbiota change by probiotic intervention

IRB No: 1507/002-012
(Institutional Review Board of Seoul National University, Korea)
Trial registration No: KCT0002008
(Clinical Research Information Service, Republic of Korea)

Principal Investigator: Heebal Kim
Tel: +82-2-880-4803,
Fax: +82-2-883-8812,
E-mail : heebal@snu.ac.kr

Seoul National University

Table of Contents

1. Statement of compliance 3
2. Protocol summary 4
2.1. Background 4
2.2. Contact Details & Status 4
2.3. Source of Monetary/Material Support & Sponsor Organization 4
2.4. Study summary 5
2.5. Study design 5
2.6. Study eligibility 6
2.7. Outcome Measure(s) 6
3. Study objectives and purposes 7
3.1. Study Purpose 7
3.2. Study background 7
4.1. Study Period 10
4.2. Study method 10
4.3. Actual study process 10
5. Study enrollment and withdrawal 11
5.1. Standard of selection 11
5.2. Standard of exception 11
5.3. Target recruitment number and grounds for choosing the sample size ... 11
6. Study detail 12
6.1. Probiotic dose 12
6.4. Variable for effect evaluation, method and interpretation (if applicable) 12
6.5. Analysis principle and method 12
6.6. Statistical analysis 13
7. Study schedule 14
8. Assessment of safety 15
8.1. Safety evaluation standard, evaluation method, and report process 15
8.2. Contract for compensation of damage 15
8.3. Ethics statement for study (Personal information of participants, Declaration of Helsinky, etc.): 15
8.4. scope of collected personal information 15
8.5. Data management-storage 16
8.6. Regards and care for the participants' safety (protocol to abort and handle side effects etc.) 16
9. Reference 17

1. Statement of compliance

This study was performed in accordance with the Institutional Review Board of Seoul National University (Korea; SNU IRB No.1507/002-012). The study was approved by the Ethical Committee of the SNU IRB (Seoul, Korea), and informed consent was obtained from all the 21 volunteers before enrollment in the study. Sampling and all subsequent steps described in the Materials and Methods have been conducted in accordance with the approved guidelines. In addition, clinical trial was retrospectively registered (2016-08-12) and approved in Clinical Research Information Service (CRIS) (KCT0002008).

2. Protocol summary

2.1. Background

Scientific Title	Identification of gut microbiota change by probiotic intervention			
CRIS Registration No.	KCT0002008			
Investigate	No	Registered at Other Registry	No	

2.2. Contact Details & Status

Contact Person for Principal Investigator / Scientific Queries	Heebal Kim	Seoul National University			
Study Center	Single	Participating Institute Name	Seoul National University		
Overall Recruitment Status	Completed	Primary Completion Date	2015-12-20	Study Completion Date	2016-06-30
Target Sample Size	21	Date of First Enrollment	2015-10-03	State of First Enrollment	Actual

2.3. Source of Monetary/Material Support & Sponsor Organization

Source of Monetary /Material Support 1	Seoul National University
Sponsor Organization 1	Seoul National University

2.4. Study summary

Study summary	While a wide variety of probiotic studies were performed, only a few attempted to simultaneously characterize interaction effects among microbiomes, host phenotypes, and probiotic intervention in human. The aim of this study was to investigate which of the gut microbes respond to probiotic intervention, composed of Lactobacilli and Bifidobacteria, as well as whether they are associated with gastrointestinal symptoms in an adult human. Twenty-one healthy adults were recruited and received a probiotic mixture, which is composed of five Lactobacilli strains and two Bifidobacteria strains, once a day for 60 days. Defecation survey and Bioelectrical Impedance Analysis were conducted before and after administration to measure phenotypic differences. Stool samples of the subjects were collected twice (before and after 60 days of probiotic administration), and metagenome analysis was performed.

2.5. Study design

Study Type	Interventional Study	Primary Purpose	Others	Phase	PhasePhase
Intervention Model	Single Group	Blinding/ Masking	Open	Allocation	Not Applicable
Intervention Type	Dietary Supplement	Number of Arms	1		

Intervention Description		All subjects received a probiotic mixture (PROBA®, 525 mg) once a day for 60 days, obtained from CTC Bio Co., Ltd., Seoul, Korea. The probiotic mixture contained 20 billion viable lyophilized bacteria and consisted of five strains of Lactobacilli (L. plantarum CLP0611, L. plantarum LP-115, L. salivarius LS-33, L. casei KE-99, and L. acidophilus LA-14) and two strains of Bifidobacteria (B. animalis subsp . lactis BL-04 and B. bifidum BB-02).				
Arm 1	Arm Label	Probiotics intervention group	Target Sample Size	21	Arm Type	experimental
	Arm Description	All subjects received a probiotic mixture (PROBA®, 525 mg) once a day for 60 days, obtained from CTC Bio Co., Ltd., Seoul, Korea. Participants took one pill of the proboitics per day				

2.6. Study eligibility

Condition(s) / Problem(s)	* Not Applicable Healthy Volunteers			Rare Disease	No
Gender	Both	Age	19 ~ 65(Years old)	Accepting Healthy Volunteers	Yes
Inclusion Criteria	Men and women between 19-65 years of age				
Exclusion Criteria	No exclusion criteria because probiotics is dietary Supplement				

2.7. Outcome Measure(s)

Type of Primary Outcome	Not applicable			
Primary Outcome 1	Outcome	Abundances of the gut microbiomes derived from feces	Timepoint	Before and after trials in 60 days probiotics intervention
Secondary Outcome 1	Outcome	Bioelectrical Impedance Analysis and Survey of gastrointestinal symptoms	Timepoint	Before and after trials in 60 days probiotics intervention

3. Study objectives and purposes

3.1. Study Purpose

This study uses cluster analysis of the gut microbes from the stool samples of office workers. Individual differences in gut microbiome composition and its reaction to probiotic mixture were analyzed. In addition, answers to dietary pattern and self-health related questionnaire were used to analyze the relationship between diet, self-health, and changes in gut microbiome composition.

3.2. Study background

Gut microbiome colonies play key roles in the intestinal canal (i.e. nutrition digestion and absorption, metabolism, defense mechanisms, etc.), which is composed of 80% of unknown bacteria (Turnbaugh et al., Nature, 2006).

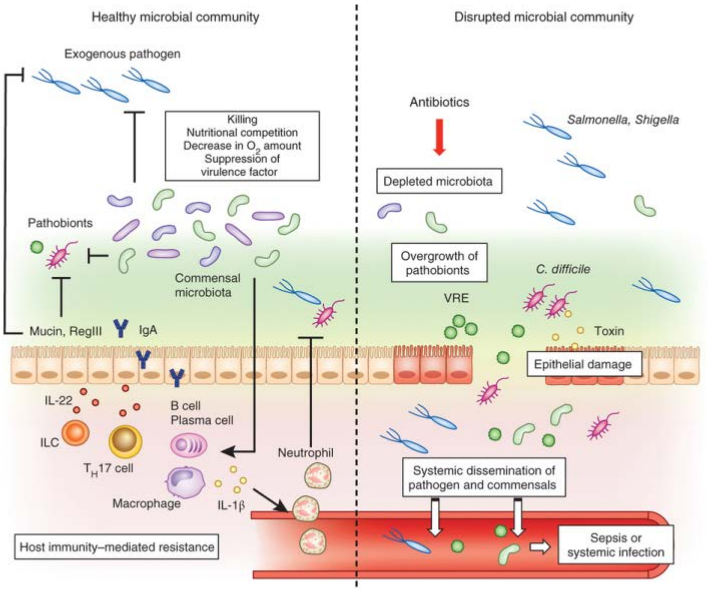

Figure 1. Gut microbiome suppression mechanism of Exogenous pathogens (Nature Immunology, 2013)

Especially, top tier journals such as Nature and Science published several works on NGS-based metagenome analysis of the gut microbiome. These works handle issues in metabolism, somatotype change, health, and growth, in relation to the gut

microbiome composition.

Even if two individuals are genetically identical, identical twins, somatotype differences between the two have been reported to be due to the gut microbiome differences (Turnbaugh et al., PNAS 2010, Ridaura et al., Science 2013). Such results display high correlation between gut microbiome and the host's body (health, etc.).

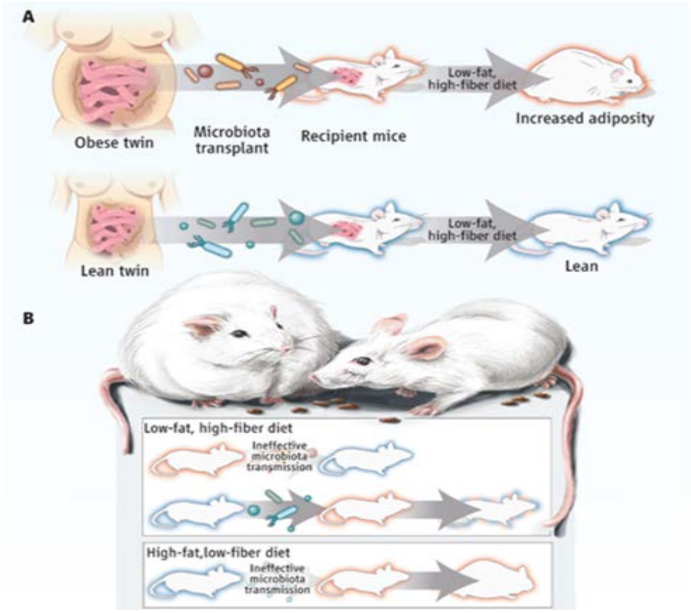

Figure 2. Fighting obesity with bacteria (Science, 2013)

So far, the gut microbiome analyses were performed by culture-dependent methods that is done by smearing the cell culture medium with the specimen and cultivating. However, such method only provides fragmentary proportion of the whole gut microbiome. In addition, culture-independent methods, such as DDGE or RT-PCR, shares the same issue of portraying only a small proportion of the whole gut microbiome. Previous works, on investigating the relationship between probiotic mixtures and gut microbiome composition, had limitations on showing the high level interactions—dynamic correlation, detailed microbiome change—through DDGE and RT-PCR analysis of stool microbiome culture analysis. This has been recently improved by NGS-based microbiome cluster analysis.

The human gut microbiome is composed of 100 trillion, >2000 in species, of bacteria. This is why the underlying mechanisms of the gut microbiome draws global attention of researchers. An extensive support from NIH through a project called “Human Microbiome Project (HMP)”, announced the microbiome genome map and database as a result of their 1st phase (2007-2012), and the 2nd phase (2013-2015) is actively on going. Additionally, an international consortium called “The International Human Microbiome Consortium (IHMC)” has been constituted to share works of microbiome researchers globally.

To measure the effects of probiotic mixtures in the gut microbiome, 20 ulcerative colitis patients were tested two times, two months and a year, after probiotic intervention. The microbiome composition of the two different periods showed high consistency (Venturi et al., 1999 Aliment Pharmacol Ther.)

Table 1. Effect of VSL#3 on intestinal flora. Modification of intestinal microflora

	Bacterial count (log 10 CFU/g faecal dry weight ± standard deviation)					
Microbiological group	Time 0	20 days	60 days	90 days	12 months	15 days after the withdrawn
Enterococci	6.79 ± 0.82	6.69 ± 0.82	6.97 ± 0.94	6.94 ± 0.59	6.90 ± 0.51	7.06 ± 0.12
S. thermophilus	< 3	7.88 ± 0.95*	8.61 ± 0.66*	8.53 ± 0.28†	8.51 ± 034	< 3
Coliforms	6.67 ± 0.76	6.97 ± 0.98	7.22 ± 0.52	6.79 ± 0.69	6.89 ± 058	6.41 ± 0.21
Total Aerobic	7.53 ± 0.67	8.11 ± 0.92	8.36 ± 0.61	8.34 ± 0.27	8.32 ± 0.35	7.82 ± 0.37
Total Anaerobic	9.45 ± 0.49	10.0 ± 0.56	9.87 ± 0.52	9.92 ± 0.23	9.98 ± 0.62	9.65 ± 0.34
Bifidobacteria	8.14 ± 0.74	9.58 ± 0.43†	9.60 ± 0.40‡	9.46 ± 0.42*	9.51 ± 0.48*	8.45 ± 0.15
Lattobacilli	4.59 ± 1.81	6.49 ± 0.97*	6.94 ± 0.49*	6.68 ± 0.91*	6.49 ± 0.65*	4.63 ± 0.72
Cl. perfrigens	5.11 ± 0.81	5.59 ± 1.10	5.94 ± 1.04	5.52 ± 1.02	5.45 ± 0.81	4.62 ± 0.07
Bacteroides	6.70 ± 1.01	7.18 ± 0.76	6.82 ± 0.66	6.57 ± 0.71	6.67 ± 0.68	7.07 ± 0.50

*$P < 0.05$; †$P < 0.01$ ‡ $P < 0.001$.

Table 1. Effects of probiotic mixture (VSL#3) on intestinal flora and modification of intestinal microflora.

A total of 10 patients, with Irritable bowel syndrome and functional diarrhea syndrome, showed improvements in their symptoms and change in microbiome composition due to probiotic intervention (Brigidi et al., 2001 Res. Microbiol.). Probiotic intervention for microbiome control and regulation have been recently highlighted, and related techniques are advancing at a high rate.

4. Study design and endpoints

4.1. Study Period

Total study period 2015 August 1st ~ 2016 June 30th

Data collection period 2015 August 15th ~ 2015 December 30th

4.2. Study method

Applicant recruitment Process: Recruit adults of age 19-65 years

Approval Process: Accept voluntary applicants only

4.3. Actual study process

The researcher provides approved (for sale) probiotics mixture, for the following two months, to the study participants. The participant takes the probiotic mixture for two months, one pill per day and without restriction of where. The participant records meals for three days prior to stool collection. He/she would use the provided stool kit twice; once in the beginning of the study and once after probiotic intervention. The researcher will visit the participant at work on the day of stool collection (twice per participant) to retrieve the stool sample along with the prior meal records (for three days). The stool samples are kept frozen until analysis. The participant is measured in the beginning of study and after intervention for height, weight, muscle mass, BMI (Body Mass Index), PBF (Percent Body Fat), WHR (Waist Hip Ratio), BMR (Basal Metabolic Rate), visceral fat, and physical development, through InBody230 of Inbody Co. And completes a 30-minute survey on dietary habit and defecation habit. To measure the effects of probiotic mixture intervention on the gut microbiome, a paired test is employed. ANOVA and linear regression is employed to investigate the correlation between the gut microbiome, inbody measurements, and survey answers.

5. Study enrollment and withdrawal

5.1. Standard of selection

Health adult of age 19 - 65

5.2. Standard of exception

Reject those who take any daily dose of antibiotics

(temporary usage for cold is acceptable)

5.3. Target recruitment number and grounds for choosing the sample size

This study's recruitment target was set based on the published work that uses 20 ulcerative colitis patients to measure the effects of probiotic mixtures in the gut microbiome (Venturi et al., 1999). This study investigated the effect of probiotic mixture at three time points; before intervention, 2-month intervention, and a year of intervention. We recruited our participants in accordance with Venturi's protocol, and collected samples from 20~25 individuals, in case of failure to collect stool samples from any of the individuals.

Group to compare against (if applicable): Not applicable

Randomization (if applicable): Not applicable

Blind method (if applicable): Not applicable

6. Study detail

6.1. Probiotic dose

Dose (Probiotic mixture one pill per day), Administration method (oral administration with water), Administration period and grounds for the term (2 month, 2 month and 1 year showed almost identical change in gut microbiome composition in the previous work (Venturi et al., 1999))

6.2. Control drug and reasoning (if applicable)

Not applicable

6.3. Observed features and method

The participant is measured in the beginning of study and after intervention for height, weight, muscle mass, BMI (Body Mass Index), PBF (Percent Body Fat), WHR (Waist Hip Ratio), BMR (Basal Metabolic Rate), visceral fat, and physical development, through InBody230 of InBody Co. And The stool samples are sequenced through 16s rRNA amplicon (V3-V4 variable region amplicon -> Miseq based 2x300bp overlapped reads) for quantitative analysis.

6.4. Variable for effect evaluation, method and interpretation (if applicable)

All the InBody measurements and additional survey are compared through a paired test.

6.5. Analysis principle and method

Measure the probiotic mixture effects on gut microbiome composition and health related index through the statistical analysis between before and after intervention. Statistical analysis will be performed through paired t-test in R.

6.6. Statistical analysis

- Subject meta data and dietary questionnaires

Subjects were asked to record all nutritional intakes for the three days before stool collection. The following six gastrointestinal symptoms were asked to the subjects before and after administration: constipation, diarrhea, irregular bowel movement, incomplete bowel movement, flatulence, and abdominal pain. The severity of symptoms are graded on a six-step scale ranging from the number (1), minimal (2), mild (3), moderate (4), severe (5), very severe, to (6) distress. To statistically test whether questionnaires' responses differ between before and after trials, ordered logistic regression model was employed to consider ordinal response variable. Symptom relief by 60 days probiotic intervention was statistically tested. In order to consider the paired sample design, ID term was included as explanatory variable along with three other covariates: Sex, height, and age. The statistical test was performed using *polr* function implemented in the MASS package of R. The square matrix was optimized via the Hessian method.

-Bioelectrical Impedance Analysis for investigation of probiotic effects in obesity indexes

A total of 11 indexes were measured by the bioelectrical impedance body composition analyzer (Inbody230, InBody Co. Ltd., Seoul, Korea): weight, skeletal muscle mass, body fat mass, total body water, fat-free mass, protein, mineral, body mass index (BMI), body fat percentage, waist-hip ratio, and basal metabolic rate. These indexes were measured three times in each before and after trials to consider technical errors in statistical analysis. In order to investigate the changes between before and after trials, measured indexes were statistically analyzed through the Analysis of Covariance

(ANCOVA) model. In addition, the three covariates: Sex, height, and age, were considered in the model for their potential effects on the measured indexes.

- Statistical analysis to detect significantly changed OTUs between before and after trials

We statistically analyzed the samples to detect the probiotic intervention effects on microbes. Before comparing OTU's abundances between before and after trials, trimmed mean of M values (TMM) normalization was performed in each taxonomic count data to consider different library size44. Using these relative abundances in each OTU, the Analysis of Deviance (ANODEV) model was employed for significance test between trials in genus, family, and phylum levels, respectively. Paired design sample was considered in this study, therefore paired test was performed. To consider the paired sample design in the model, the 'Individual' term was included as an explanatory variable in the linear predictor as shown in (Eq. 4). Finally, the negative-binomial assumption was considered as a response variable to solve the over-dispersion problem in count data. Under the null hypothesis, $H_0\colon Stage = 0$, likelihood ratio test (LRT) was performed and probability values were adjusted by false discovery rate (FDR) multiple testing adjustment. Here, 5% significance level was considered as significant result.

7. Study schedule

Estimated Duration	Schedule
2015/05/29 – 2015/6/15	IRB application and review
2015/06/16 – 2015/07/15	Begin study, Applicant recruitment
2015/07/01 – 2015/12/31	Data collection (probiotic mixture consumption for 2 month)
2016/01/01 – 2016/12/31	Data analysis and manuscript submission
	6 month ~ 1 year until publication

8. Assessment of safety

8.1. Safety evaluation standard, evaluation method, and report process

The probiotic mixture (PROBA For Family) has been approved by the Ministry of Food and Drug Safety as a health functional food for sale (filed for import declaration and approved).

8.2. Contract for compensation of damage

The probiotic mixture is a health functional food that is approved for sale, and the researchers do not expect any side effects.

8.3. Ethics statement for study (Personal information of participants, Declaration of Helsinky, etc.):

This study was performed in accordance with the Institutional Review Board of Seoul National University (Korea; SNU IRB No.1507/002-012). The study was approved by the Ethical Committee of the SNU IRB (Seoul, Korea), and informed consent was obtained from all the 21 volunteers before enrollment in the study. This study's recruitment target was set based on the published work that uses 20 ulcerative colitis patients to measure the effects of probiotic mixtures in the gut microbiome (Venturi et al., 1999). We recruited our participants in accordance with Venturi's protocol, and collected samples from 20~25 individuals, in case of failure to collect stool samples from any of the individuals.

8.4. scope of collected personal information

Name, Age, Gender, Contact, InBody measurements (height, weight, muscle mass, BMI (Body Mass Index), PBF (Percent Body Fat), WHR (Waist Hip Ratio), BMR (Basal Metabolic Rate), visceral fat, and physical development), survey daily antibiotics administration information, dietary habit, smoking status, and self-evaluation indexes on bowel health, immunity, chronic fatigue syndrome

8.5. Data management-storage

Principal investigator holds the password for the document folder in a separate computer.

8.6. Regards and care for the participants' safety (protocol to abort and handle side effects etc.)

The probiotic mixture is approved for sale, as health functional food, yet if the participants show any sign of side effects, the study should be aborted and the participant should contact the researcher immediately. If the side effect is severe, immediate medical care is to be given by a doctor. If any of the participant refuses to continue at any time of the study, the researcher should immediately discard all records and personal information of the patient.

9. Reference

Turnbaugh P, Ley R, Mahowald M, Magrini V, Mardis E & Gordon J. 2006. An obesity-associated gut microbiome with increased capacity for energy harvest. Nature. 444:1027-1031

Kamada N Chen G, Inohara N & Nunez G. 2013. Control of pathogens and pathobionts by the gut microbiota. Nature Immunology. 14:685-90

Turnbaugh P, Quince C, Faith J, McHardy A, Yatsunenko T, Niazi F, Affourtit J, Egholm M, Henrissat B, Knight R, Gordon J. 2010. Organismal, genetic, and transcriptional variation in the deeply sequenced gut microbiomes of identical twins. Proc. Natl. Acad. Sci. 107:7503-7508.

Ridaura V. et al. 2013. Gut microbiota from twins discordant for obesity modulate metabolism in mice. Science. 341:1241214.

Venturi A, Gionchetti P, Rizzello F, Johansson R, Zucconi E, Brigidi P, Matteuzzi D & Campieri M. 1999. Impact on the composition of the faecal flora by a new probiotic preparation: preliminary data on maintenance treatment of patients with ulcerative colitis. Aliment Pharmacol Ther. 13(8):1103-8.

Brigidi P, Vitalia B, Swennena E, Bazzocchib G, Matteuzzia D, 2001. Effects of probiotic administration upon the composition and enzymatic activity of human fecal microbiota in patients with irritable bowel syndrome or functional diarrhea. Res. Microbiol. 152:735-741

Tannock, G. W., et al. "Analysis of the fecal microflora of human subjects consuming a probiotic product containing Lactobacillus rhamnosusDR20." Applied and environmental microbiology 66.6 (2000): 2578-2588.

Birkett, Anne, et al. "Resistant starch lowers fecal concentrations of ammonia and phenols in humans." The American journal of clinical nutrition (1996): 766-772.
